# Quantification of cysteine hydropersulfide with a ratiometric near-infrared fluorescent probe based on selenium–sulfur exchange reaction†
†Electronic supplementary information (ESI) available: Experimental detail procedures, synthetic procedures and characterization details, reaction kinetics and selectivity, and additional data. See DOI: 10.1039/c6sc00838k


**DOI:** 10.1039/c6sc00838k

**Published:** 2016-04-11

**Authors:** Xiaoyue Han, Fabiao Yu, Xinyu Song, Lingxin Chen

**Affiliations:** a Key Laboratory of Coastal Environmental Processes and Ecological Remediation , Yantai Institute of Coastal Zone Research , Chinese Academy of Sciences , Yantai 264003 , China . Email: lxchen@yic.ac.cn; b University of Chinese Academy of Sciences , Beijing 100049 , China

## Abstract

Cysteine hydropersulfide (Cys-SSH) plays primary roles in the synthesis of sulfur-containing cofactors, regulation of cellular signaling, activation or inactivation of enzyme activities, and modulation of cellular redox milieu. However, its biofunctions need to be further addressed due to the fact that many issues remain to be clarified. Herein, we conceive a novel ratiometric near-infrared fluorescent probe **Cy-Dise** for the sensitive and selective detection of Cys-SSH in living cells and *in vivo* for the first time. **Cy-Dise** is composed of three moieties: bis(2-hydroxyethyl) diselenide, heptamethine cyanine, and d-galactose. **Cy-Dise** exhibits a satisfactory linear ratio response to Cys-SSH *via* a selenium–sulfur exchange reaction in the range of 0–12 μM Cys-SSH. The experimental detection limit is determined to be 0.12 μM. The results of ratio imaging analyses confirm the qualitative and quantitative detection capabilities of **Cy-Dise** in HepG2 cells, HL-7702 cells, and primary hepatocytes. The level changes of Cys-SSH in cells stimulated by some related reagents are also observed. The probe is also suitable for deep tissue ratio imaging. Organ targeting tests with **Cy-Dise** in normal Spraque-Dawley (SD) rats and Walker-256 tumor SD rats verify its predominant localization in the liver. The probe is promising for revealing the roles of Cys-SSH in physiological and pathological processes.

## Abstract 2

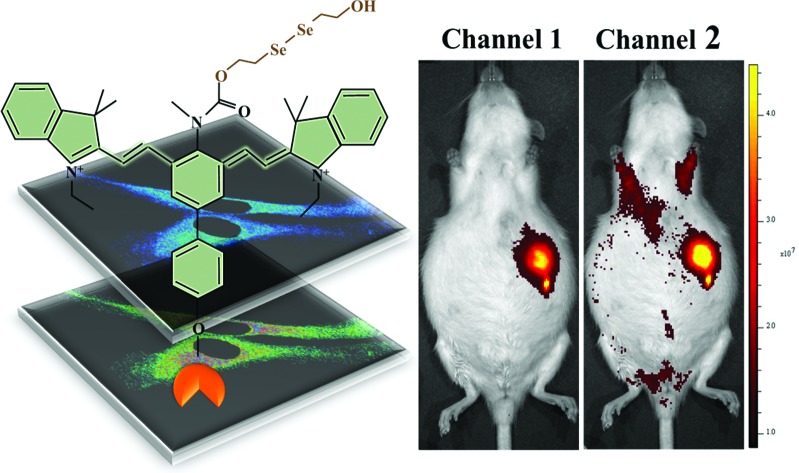
A ratiometric near-infrared fluorescent probe based on a selenium–sulfur exchange reaction to quantify cysteine hydropersulfide in living cells and hepatic carcinoma rats.

## Introduction

The chemical flexibility of sulfur has led to its wide utilization in sulfur-containing biomolecules which are known as reactive sulfur species (RSS).^1^ RSS exist in all kinds of cells and tissues, and play pivotal roles in many physiological processes, such as antioxidants and signal transduction.^2^ The RSS which are involved in physiological processes are often together with the trafficking and delivery of sulfur in protein cysteine residues (called *S*-sulfhydration).^3^ The dysregulation of *S*-sulfhydration in protein cysteine residues will cause undesired changes to protein functions and lead to many diseases such as atherosclerosis, hypertension, diabetes and strokes, cancer, and neurodegeneration.^4^ Since the hydropersulfide group (R–S–SH) predominantly serves as the sulfur donor, the simple cysteine hydropersulfide (Cys-SSH) may contribute to the primary role in providing sulfur for cofactors, modulating cellular signaling, and activating or inactivating enzyme activities.^5^ Moreover, Cys-SSH can modulate the cellular redox milieu by increasing the reductive capacity of glutathione (GSH).^5^ GSH plays crucial roles in antioxidant defense. However, the GSH associated with antioxidant activities are typically mediated by specific enzymes, such as GSH-dependent peroxidase and glutathione *S*-transferase.^6^ Without the participation of enzymes, GSH is inert to electrophilic oxidants.^7^ Meanwhile, glutathione persulfide (GSSH), the main Cys-SSH derivative, can be employed as a direct and potent antioxidant in cells. The levels of GSSH can rise to 100 μM in the cells and tissues of mice.^8^ It is noteworthy that the generation of Cys-SSH in biosystems results in the subsequent formation of Cys/GSH-based hydropersulfides (*e.g.* GSSH, Cys-SS_*n*_SH, GSS_*n*_SH, *n* > 1).^8^ Hence, Cys-SSH may be the source of the whole profile of hydropersulfide derivatives in living cells and *in vivo*.

The intracellular levels of Cys-SSH can be maintained at the micromolar level.^8^ The major production pathway of Cys-SSH is mediated by the enzymes cystathionine γ-lyase (CSE) and cystathionine β-synthase (CBS), which are often thought to generate H_2_S.^9^ Another essential biosynthetic method for Cys-SSH generation is the pyridoxal and Cu^2+^-catalyzed non-enzymatic α,β-elimination reaction of cystine.^10^ 3-mercaptopyruvate sulfurtransferase (MST) can transfer sulfur to form Cys-SSH as well.^8^,^11^ Cys-SSH can also be obtained from other pathways such as cystathionase (CST),^12^ quinone oxidoreductase (SQR),^13^ sulfurtransferase IscS,^14^ and the Cys aldimine/ketimine state.^15^ Initially, H_2_S is supposed to modify cysteine to form Cys-SSH. However, the direct reaction of H_2_S with cysteine is infeasible except in the presence of oxidant or enzyme.^4^,^16^ Recent reports imply that Cys-based persulfides rather than H_2_S may be the actual signal transduction molecules. However, in contrast to their thiol analogs (RSH),^17^ the fundamental chemistry and chemical biology of persulfides in cells is yet to be elucidated.^2^,^8^,^18^ Although detection methods for other RSS (such as H_2_S, GSH and Cys) have elegantly accumulated,^19^ methods for the detection of hydropersulfides are urgently required. Cyanolysis cannot be employed to identify hydropersulfides in complex systems like cell lysates.18*b* Additionally, 2,4-dinitrothiophenol methods are also limited to detect purified hydropersulfides.18*b* These two methods will overestimate hydropersulfides due to the interference from other thiol-alkylations. The detection methods that include modified biotin and tag-switch assays are accurate approaches.^8^ However, these methods require complicated sample pretreatment and cannot satisfy the requirements of real-time and *in situ* detection *in vivo* because of the unstable properties of hydropersulfides. Compared with other biological detection technologies, fluorescence imaging has become an essential tool for the detection of a variety of reactive species in cells, such as reactive oxygen species (ROS),^20^ reactive nitrogen species (RNS),^21^ RSS,^22^ enzymes^23^ and metal ions^24^ due to its several advantages including reduced invasiveness, rapid response, and high spatial and temporal resolution. Herein, our objective is to exploit a new chemical inspection tool for the detection of cysteine-based hydropersulfides (mainly as Cys-SSH) in intact cells.

**Scheme 1:**
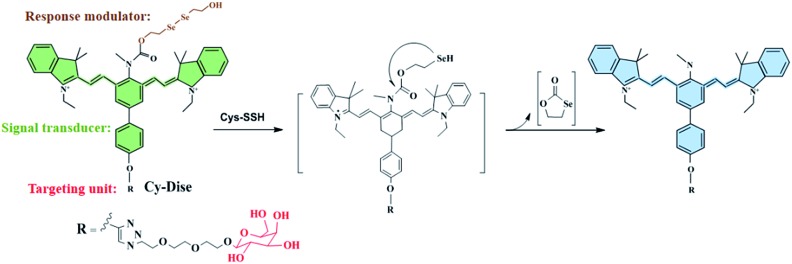
Illustration for the structure of **Cy-Dise** and the proposed selenium–sulfur exchange reaction that modulates fluorescence changes through an intramolecular cyclization reaction.

Herein, we describe a liver-targeting ratiomeric NIR fluorescent probe (**Cy-Dise**) for the selective detection of Cys-SSH in living cells and *in vivo* (Scheme 1). Once the ICT process is triggered by Cys-SSH, the probe exhibits a larger spectral blue shift. The fluorescence response of **Cy-Dise** to Cys-SSH is rapidly completed within minutes. This rapid response feature plays a crucial role in fast detection on account of the quick metabolism and unstable properties of Cys-SSH in biological systems. The test results enable the probe to qualify and quantify Cys-SSH in HepG2 cells, HL-7702 cells and primary mouse hepatocyte cells. Moreover, **Cy-Dise** preferentially accumulates in the carcinoma tissue of Walker-256 tumor-bearing rats because the transplantation model of liver cancer can overexpress ASGP-R.

## Results and discussion

### Design and synthesis of **Cy-Dise**

There is a quite limited number of fluorescent probes that have been designed and synthesized for visualizing and quantifying the overall levels of persulfides and hydrogen polysulfides in cells and *in vivo*.^25^ To the best of our knowledge, appropriate probes for the selective detection of Cys-SSH have rarely been reported.9*b* Therefore, it is extremely urgent to develop a kind of fluorescent probe which owns the ability to track Cys-SSH for researching the biofunctions of Cys-SSH in living cells and *in vivo*. However, the detection of Cys-SSH is challenging for four reasons: (1) the selective detection of persulfides is seriously affected by endogenous thiols, such as GSH whose concentration ranges from 1 to 15 mM depending on the cell types;^7^,^26^ (2) the biofunctions of persulfides have only been recently established and there are few illustrations of accurate protein persulfides models;^27^ (3) the persulfide species have high reactivity, and exhibit a short lifespan in aerobic conditions; and (4) interferences from coexisting nitrosothiol (RSNO) and sulfenic acid (RSOH) species are difficult to avoid due to their strong electrophile properties.^1^,^18^,^28^

Our design concept is inspired by the significantly different p*K*_a_ between Cys-SSH and its structurally related thiol analogs. The p*K*_a_ of Cys-SSH is approximately 4.34, which makes it more strongly acidic compared to Cys (p*K*_a_ = 8.29) and GSH (p*K*_a_ = 8.75).^29^ At physiological pH (7.40), Cys-SSH predominantly exists as the deprotonated anion (Cys-SS^–^), while other biothiols exist as the protonated forms. Therefore, Cys-SSH has stronger nucleophilic properties than Cys and GSH. For conceiving the new probe, we first smartly introduce the reactive diselenide (R–Se–Se–R) as the Cys-SSH response modulator. It is promising that the diselenide can be rapidly reduced owing to the stronger nucleophilic properties of Cys-SSH over other biothiols.^30^ Moreover, the satisfactory response unit can successfully avoid the interferences from RSNO and RSOH, because these two kinds of compounds cannot exhibit reactivity towards the diselenide bond. The increase or decrease of fluorescent probes which exhibit one intensity-responsive signal can be interfered with by the excitation and emission efficiency.^31^ Fortunately, ratiometric probes which employ the ratio of the spectra at two or more emission bands can overcome this problem. Variable factors derived from uneven loading or inhomogeneous distribution of the probes and environmental conditions can be eliminated.^32^ Ratiometric probes have been proven to be powerful tools to qualitatively and quantitatively detect biomolecules in living cells and *in vivo*.^33^,^34^ For the purpose of penetrating sufficiently into tissues and avoiding biological auto-fluorescence interference, probes which work at the near-infrared (NIR) region can meet the requirements.^35^ We choose a NIR dye, heptamethine cyanine, as the signal transducer. The facile modulation of different electron-donating donors in the middle position on the fluorophore platform will result in internal charge transfer (ICT)-induced blue or red shifts in its emission spectrum.^36^ The liver is a predominant innate immunologic and essential metabolic organ *in vivo*. Liver damage ranges from acute hepatitis to hepatocellular carcinoma whose induction and progression are close related to oxidative stress.^37^ When the liver suffers oxidative stress, the enzyme CSE will protect the sensitive proteins by *S*-sulfhydration of cysteine residues.^38^ In addition, Cys-SSH, the main product of CSE, can be carried from the liver to other organs through circulation.^12^,^39^ We suppose that a liver-targeting fluorescent probe can help illustrate the formation and function mechanism of Cys-SSH. We eventually achieve the liver-targeting hypothesis *via* introducing a galactose-terminated ligand into the fluorophore platform, since the asialoglycoprotein receptor (ASGP-R) selectively accepts the terminal galactose residues on desialylated glycoproteins,^40^ and ASGP-R specifically expresses on the plasma membrane of mammalian hepatocytes.^41^

**Scheme 2:**
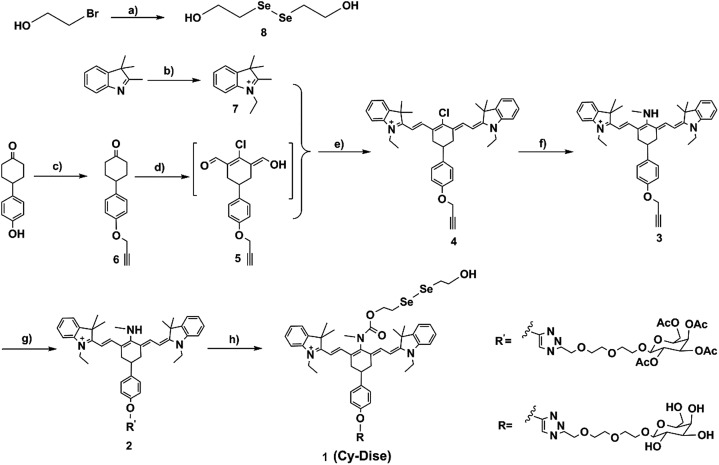
Synthetic routes for the probe **Cy-Dise** (a) NaBH_4_, H_2_O, 25 °C, 15 min, then Se, reflux 3 h; 2-bromoethanol, THF, 50 °C, 6 h, 24%. (b) CH_3_CH_2_I, acetonitrile, reflux 12 h, 80%. (c) NaH, DMF, 0 °C, 20 min, then propargyl bromide, 25 °C, 24 h, 55%. (d) DMF/CH_2_Cl_2_, –10 °C, 20 min, then POCl_3_; reflux 3 h, in ice overnight, 43%. (e) In *n*-butyl alcohol and benzene (7 : 3 v/v), reflux 3 h, 70%. (f) CH_3_NH_2_·HCl, Et_3_N, 40 °C, 24 h, 87%. (g) Sodium ascorbate/CuSO_4_, H_2_O, then MeOH, DIPEA, acetyl-d-galactopyranoside, 25 °C, 24 h, 20%. (h) Triphosgene, DIPEA, 0 °C, 3 h, then DIPEA, DMAP, (HOCH_3_CH_2_Se)_2_, CH_2_Cl_2_, 25 °C, 12 h, 87%; CH_3_ONa, CH_3_OH, 25 °C, 1 h, 61%.

As shown in Schemes 1 and 2, the new probe **Cy-Dise** is composed of three moieties: (i) response modulator: bis(2-hydroxyethyl) diselenide (Dise); (ii) signal transducer: heptamethine cyanine (Cy); and (iii) targeting unit: d-galactose. The amino-nucleophilic substitution on the middle position of the signal transducer can efficaciously modulate a large blue shift compared to its original emission spectrum. However, the integration of the response modulator with a carbonyl group into the central nitrogen atom will result in a red shift in the emission spectrum. We speculate that the electron-withdrawing group, the carbonyl, can suppress the electron density of the amino-substituent, resulting in the signal transducer recovering its emission spectrum.33b,^42^ The removal of the response modulator by Cys-SSH will lead to a blue shift of the emission spectrum again. The ICT-based red–blue shift in the emission spectrum will provide a desirable ratio signal for the detection of Cys-SSH in living cells and *in vivo*. The removal of the response modulator to release the fluorophore involves a two-step process, as illustrated in Scheme 1. The intermediate is formed immediately upon the reduction of diselenide,^30^ and then a fast intramolecular cyclization occurs by cleavage of the neighboring carbamate bond to release the fluorophore. The intermediate was confirmed by high resolution mass spectrometry (HRMS) (Fig. S6†). However, the formation of the five-membered cyclic selenocarbonate could not be detected by HRMS which might be attributed to its instability in solution.^43^

As shown in Scheme 2, the response modulator bis(2-hydroxyethyl) diselenide (**8**) was synthesized from selenium and 2-bromoethanol. The heptamethine cyanine dye (**4**) was designed with a propargyloxy group at the *para*-cyclohexylbenzene position. Propargyl bromide and 4-(4-hydroxyphenyl)cyclohexanone were used as the starting materials to provide 4-(4-(prop-2-yn-1-yloxy)phenyl) cyclohexanone (**6**). Compound **6** reacted with DMF and phosphorus oxychloride (POCl_3_) in anhydrous CH_2_Cl_2_ to yield the intermediate **5***via* the Vilsmeier–Haack reaction. The mixture of intermediate **5** and 1-ethyl-2,3,3-trimethyl-3*H*-indolenium iodide salt (**7**) in *n*-butyl alcohol and benzene (7 : 3 v/v) was refluxed for 3 h, which afforded compound **4**. After the nucleophilic substitution at the *meso*-position of **4** by methylamine hydrochloride (CH_3_NH_2_·HCl) in anhydrous DMF, we obtained compound **3**. Acetyl-d-galactopyranoside was integrated into **3***via* a click chemistry reaction to produce compound **2**. Compound **2** was next treated with triphosgene. Then the solvent was blow-dried by nitrogen stream. Subsequently, compound **8** was added to the reaction system to produce the pre-product. After hydrolyzing the acetyl groups, we finally obtained the probe **Cy-Dise**. All the details of the syntheses are described in the ESI.†

### Spectral properties and selectivity of **Cy-Dise**

**Fig. 1:**
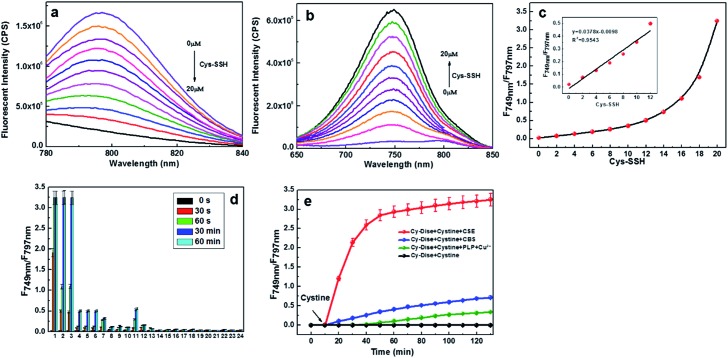
Spectral properties and selectivity of **Cy-Dise**. (a) *λ*_ex_ = 730 nm, (b) *λ*_ex_ = 614 nm. Dose-dependent emission spectra of **Cy-Dise** (10 μM) towards Cys-SSH. Date were recorded after 2 min with increasing concentrations of Cys-SSH (0–20 μM) at 37 °C in HEPES (pH 7.4, 10 mM). (c) Ratio signals (*F*_749 nm_/*F*_797 nm_) of **Cy-Dise** towards Cys-SSH. Insert: the linear relationship between the ratio signals and the concentrations of Cys-SSH (0–12 μM). (d) Ratio signals (*F*_749 nm_/*F*_797 nm_) of **Cy-Dise** (10 μM) to reactive sulfur and selenium species in HEPES (pH 7.4, 10 mM) at 37 °C: 1, 20 μM Cys-SSH; 2, 20 μM GSSH; 3, 20 μM persulfide P; 4, 60 μM human serum albumin persulfide; 5, 60 μM papain persulfide; 6, 60 μM Gpx3 persulfide; 7, 100 μM NaHS; 8, 100 μM Cys; 9, 20 μM cysteine methyl ester; 10, 40 μM Cys-polysulfide; 11, 20 μM Na_2_S_4_; 12, 20 μM Na_2_S_4_ + 40 μM Cys-polysulfide; 13, 100 μM Hcys; 14, 1 mM GSH; 15, 50 μM Cys-SS-Cys; 16, 50 μM S_2_O_3_^2–^; 17, 50 μM HSO_3_^–^; 18, 50 μM AhpC-SOH; 19, 50 μM GSSG; 20, 50 μM lipoic acid; 21, 100 μM ascorbic acid; 22, 50 μM tocopherol; 23, 50 μM metallothionein; 24, 50 μM selenocysteine. All data were obtained at 0 s, 30 s, 60 s, 30 min and 60 min. (e) Time-dependent ratio signals of **Cy-Dise** (10 μM) towards Cys-SSH catalyzed by CSE (50 μg mL^–1^), CBS (5 μg mL^–1^), and PLP (1.0 × 10^–6^ mol L^–1^) and Cu^2+^ (1.0 × 10^–6^ mol L^–1^), with cystine (1 mM) as substrate. Data were acquired in HEPES buffer (pH 7.4, 10 mM) at 37 °C for 130 min.

The absorption and fluorescence spectra of **Cy-Dise** toward Cys-SSH were measured under simulated physiological conditions (10 mM HEPES, pH 7.4, 37 °C). Upon detecting Cys-SSH, the maximum absorption changed from 790 nm (*ε*_790 nm_ = 1.02 × 10^4^ M^–1^ cm^–1^) to 614 nm, (*ε*_614 nm_ = 3.01 × 10^4^ M^–1^ cm^–1^) accompanied by a color change from green to blue (Fig. S1†). Also, the maximum emission wavelength shifted from 797 nm (*Φ*_797 nm_ = 0.05) to 749 nm (*Φ*_749 nm_ = 0.11) (Fig. 1a and b). The fluorescence intensity ratio (*F*_749 nm_/*F*_797 nm_) was positively correlated with the Cys-SSH concentration (Fig. 1c). There existed a linear relationship between the signal ratios and the concentrations of Cys-SSH from 0–12 μM (Fig. 1c insert). The regression equation was *F*_749 nm_/*F*_797 nm_ = 0.0378[Cys-SSH] μM – 0.0098, with *r* = 0.9913. The experimental detection limit was determined to be 0.12 μM. The theoretical detection limit was calculated to be as low as 57 nM (3*σ*/*k*), where *σ* is the standard deviation of the blank measurement, and *k* is the slope of the regression equation. These results indicated the potentiality of **Cy-Dise** for quantitative and qualitative ratiometric detection of Cys-SSH.

Our next efforts were made to test and verify the selectivity of **Cy-Dise** towards Cys-SSH. All the tests lasted for 60 min. Upon exposure to various analytes in HEPES (10 mM, pH 7.4), the probe **Cy-Dise** selectively exhibited an excellent ratio fluorescence response to Cys-SSH (Fig. 1d). What needs to be explained here is that the probe **Cy-Dise** would give a fluorescence response to a wide range of hydropersulfide species. However, the response kinetics depended on the intrinsic nucleophilic nature of the hydropersulfide species. Among these hydropersulfide species, Cys-SSH is the primary species. Therefore, we singled out Cys-SSH as a hydropersulfide to test. We also confirmed the detection by testing persulfide P, an analogue of the nitrosothiol *S*-nitrosoacetyl-penicillamine (SNAP). Protein persulfides, such as human serum albumin persulfide, papain persulfide and Gpx3 persulfide could not induce big interferences. Other RSS, and reducing biosubstances, including NaHS, cysteine (Cys), cysteine methyl ester, Cys-polysulfide, Na_2_S_4,_ a mixture of Na_2_S_4_ and Cys-polysulfide, homocysteine (Hcys), glutathione (GSH), oxidized glutathione (GSSG), cystine (Cys-SS-Cys), S_2_O_3_^2–^, HSO_3_^–^, metallothionein, protein sulfenic acids (AhpC-SOH), lipoic acid, ascorbic acid, and tocopherol, cannot induce interference. ROS, RNS, and anions and metal ions also do not cause any spectral changes (Fig. S4a and b†). Reactive selenium species, such as selenocysteine, also could not trigger interference. All the results demonstrated that **Cy-Dise** could selectively detect Cys-SSH without interference by other species.

It is suggested that Cys-SSH can be biosynthesized from enzyme and non-enzyme approaches.9a,^12^ We tried to examine whether **Cy-Dise** could detect Cys-SSH that was catalyzed by CSE, CBS, and pyridoxal-phosphate (PLP). The substrate of the three approaches was Cys-SS-Cys. The catalyzed reaction of PLP required an auxiliary by Cu^2+^. As shown in Fig. 1e, all the three approaches could offer Cys-SSH and yield time-dependent fluorescence responses. However, only the catalysis of CSE could trigger a fast increase in the ratio signal. The result implied that CSE might be the major biological pathway for the direct generation of Cys-SSH.9*a*

### Quantification of endogenous Cys-SSH in living cells

Since the probe **Cy-Dise** had exhibited good sensitivity and selectivity towards Cys-SSH under simulated physiological conditions, we further tested the potential utility of **Cy-Dise** for fluorescence imaging of Cys-SSH in living cells and *in vivo*. We selected HL-7702 cells (human normal liver cell line), HepG2 cells (human hepatocellular liver carcinoma cell line), and primary mouse hepatocyte cells (female BALB/c mice) as test models to evaluate the formation and intracellular concentrations of Cys-SSH. Prior to cell tests, MTT assays were performed to check the cytotoxicity of **Cy-Dise**. The high cell viability of **Cy-Dise** indicated that the probe displayed low cytotoxicity to living cells (Fig. S8†).

**Fig. 2:**
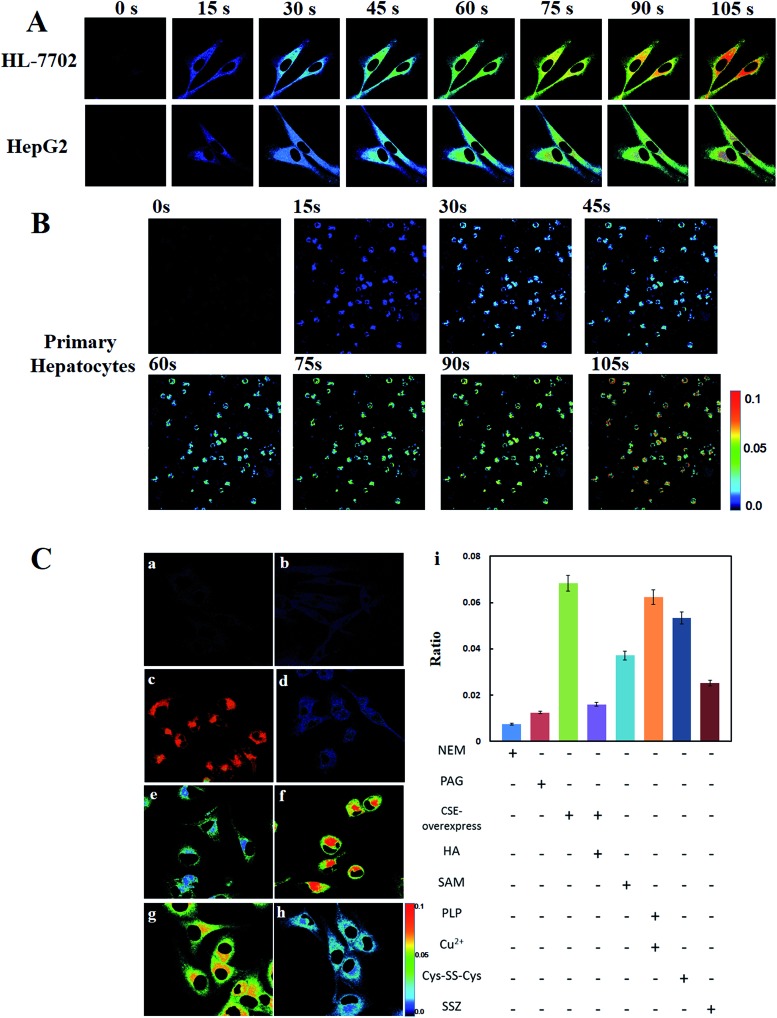
Time-dependent ratio images (*F*_channel 2_/*F*_channel 1_) of endogenous Cys-SSH analyses in living cells. (A) HepG2 cells and HL-7702 cells; (B) primary hepatocytes. Cells were incubated with 10 μM **Cy-Dise**, and then ratio images were recorded at different time points: 0 s, 15 s, 30 s, 45 s, 60 s, 75 s, 90 s and 105 s. Pseudo-color ratio images indicate the ratio of channel 2 *vs.* channel 1 at corresponding time points. Fluorescence collection windows for channel 1: 750–800 nm (*λ*_ex_ = 730 nm), channel 2: 690–740 nm (*λ*_ex_ = 635 nm). Scale bar = 10 μm. (C) Ratio images of the endogenous Cys-SSH generation in living HL-7702 cells exposed to different stimulation agents. All the cells were stained by 10 μM **Cy-Dise** for imaging. (a) Cells incubated with 5 mM NEM for 30 min; (b) cells incubated with 100 μM PAG for 30 min; (c) imaging of CSE-overexpress cells; (d) CSE-overexpress cells incubated with 250 μM HA for 30 min; (e) cells incubated with 3 mM SAM for 1 h; (f) cells incubated with 1 μM pyridoxal phosphate and 1 μM copper ion for 6 h; (g) cells incubated with 100 μM Cys-SS-Cys for 1 h; (h) imaging of cells incubated with 100 μM SSZ for 3 h. Pseudo-color ratio images indicate the ratio of channel 2 *vs.* channel 1 at the same time point. Fluorescence collection windows for channel 1: 750–800 nm (*λ*_ex_ = 730 nm), channel 2: 690–740 nm (*λ*_ex_ = 635 nm). Scale bar: 10 μm. (i) Quantitative application of **Cy-Dise** by flow cytometry analysis (*n* = 3). The graph shows the ratio of the mean fluorescence intensities of two different detection channels corresponding in (a)–(h).

Cell imaging experiments were performed by utilizing laser scanning confocal microscopy. All the three groups of testing cells in Fig. 2 were incubated with 10 μM **Cy-Dise** for 5 min before imaging. The ratiometric fluorescence imaging was constructed *via* two fluorescence collection windows, that is, channel 1 from 750 to 800 nm (*λ*_ex_ = 730 nm), and channel 2 from 690 to 740 nm (*λ*_ex_ = 635 nm). As shown in Fig. 2A, time-dependent ratio fluorescence responses to Cys-SSH were surveyed during the period 0–105 s. The ratio intensity of the HL-7702 and HepG2 cells gradually increased during the testing time (also shown in ESI Movies 1 and 2†). However, the different changing speeds obviously demonstrated the distinct endogenous Cys-SSH-producing capabilities between HL-7702 and HepG2 cells. We chose the time point at 105 s to determine the concentrations of Cys-SSH in the two types of cells. *F*_channel 1/channel 2_ = 0.0601 for the HL-7702 cells, and *F*_channel 1/channel 2_ = 0.0204 for the HepG2 cells. After calculations using the regression equation in Fig. 1c, we obtained concentrations of Cys-SSH of 1.85 ± 0.2 μM and 0.80 ± 0.1 μM in HL-7702 and HepG2 cells, respectively. Flow cytometry analysis is considered to be a technology that allows rapid analysis of millions of cells and generates statistically convincing data. To further confirm the ratio image changes in HL-7702 and HepG2 cells caused by Cys-SSH, we performed a flow cytometry assay to test and verify the results in Fig. 2A. The cells were treated as described in Fig. 2A (*n* = 3). As shown in Fig. S11A,† the mean fluorescence intensity decreased in channel 1 and simultaneously increased in channel 2 during the period 0–105 s. *F*_channel 1/channel 2_ = 0.0616 for HL-7702 cells, and *F*_channel 1/channel 2_ = 0.0212 for HepG2 cells at the time point 105 s (Fig. S11B†). The concentrations of Cys-SSH in HL-7702 and HepG2 cells were determined to be 1.89 ± 0.2 μM and 0.82 ± 0.3 μM, respectively. There was no doubt that the two testing results were consistent with each other. Additionally, we exploited a Tag-Switch assay to reinforce the above results. Cys-SSH was readily labelled by monobromobimane (Br-bimane). The Tag-labelled Cys-SSH could be accurately analyzed by means of LC-MS/MS.^8^ We obtained the concentrations of Cys-SSH of 1.94 ± 0.4 μM and 0.88 ± 0.2 μM in HL-7702 and HepG2 cells, respectively (Fig. S16†). It was encouraging that the results of the ratio images, flow cytometry analysis, and LC-MS/MS were close, which indicated the further potential applications of our probe in living cells.

Having known that the probe **Cy-Dise** could act as a promising imaging tool for the detection of Cys-SSH in HL-7702 and HepG2 cells, we further examined its applications for the quantitative detection of Cys-SSH in primary mouse hepatocyte cells. Primary hepatocytes were exploited from female BALB/c mice. Then the cells were set in Petri dishes for 1.5 h to be adherent for imaging. As displayed in Fig. 2B, the ratio rapidly increased during the test duration. The ratio response was 0.0851 (*F*_channel 1/channel 2_) at the time point 105 s, and the corresponding concentration of Cys-SSH was determined to be 2.51 ± 0.4 μM in primary hepatocytes. The concentrations were 2.55 ± 0.2 μM from the flow cytometry analysis, and 2.58 ± 0.3 μM from LC-MS/MS. Taken together, the probe **Cy-Dise** was proved to effectively qualitatively and quantitatively analyze Cys-SSH in living cells.

### Imaging of Cys-SSH fluctuations in cells

Stimulating the endogenous Cys-SSH generation systems would disturb the levels of Cys-SSH in living cells. We next applied the probe **Cy-Dise** to investigate the perturbation for Cys-SSH biosynthetic pathways. As shown in Fig. 2C, HL-7702 cells were divided into eight groups. All the parallel groups were incubated with 10 μM **Cy-Dise** for 5 min. Then the imaging tests lasted for 60 s. The cells in the first group (Fig. 2C-a) were pretreated with *N*-ethylmaleimide (NEM) to deplete all the endogenous Cys-SSH. There was nearly no ratiometric fluorescence signal observed. The CSE-mediated conversion of Cys-SS-Cys to Cys-SSH may be the major pathway of biological persulfide generation.9*a* Pretreatment of the cells in the second group (Fig. 2C-b) with dl-propargylglycine (PAG), a CSE inhibitor,^44^ also gave a low ratio response indicating that the enzymatic activity of CSE was inhibited. The results also demonstrated that our probe **Cy-Dise** could selectively respond to Cys-SSH avoiding other biothiols. Subsequently, the enzyme CSE was overexpressed in the third group. The cells in Fig. 2C-c showed a strong increase in ratio response, which revealed a high level of Cys-SSH in the cells. However, the CSE-overexpress cells in the fourth group were treated with hydroxylamine (HA) to inhibit CSE activity.^44^ The ratio imaging in Fig. 2C-d exhibited a low level of Cys-SSH.

Next, **Cy-Dise** was applied to image CBS enzyme-mediated Cys-SSH biosynthesis. The fifth group cells were stimulated with *S*-adenosyl-l-methionine (SAM)^45^ to induce the activity of CBS. The results of Fig. 2C-e showed the weaker ability of CBS than CSE for producing Cys-SSH. The non-enzymic α,β-elimination reaction of cysteine by pyridoxal and copper ions also can produce Cys-SSH.^46^ The sixth group cells were pretreated with1 μM pyridoxal phosphate and 1 μM copper ions for 6 h. Fig. 2C-f illustrates the ratiometric image for the detection of Cys-SSH. The results indicated that pyridoxal phosphate and copper ions could generate Cys-SSH in cells. As is known, the major enzyme substrate for the generation of Cys-SSH was cystine. Therefore, the level of intracellular cystine should be an important issue for endogenous Cys-SSH generation. The last two group cells were set to inspect this point. The cells in Fig. 2C-g were pretreated with 100 μM cystine for 1 h, and then the level of Cys-SSH was evaluated with **Cy-Dise**. The cysteine/glutamine transporter (xCT) is one of the transporters of cystine, and xCT can be inhibited by sulfasalazine (SSZ).^8^ The cells in Fig. 2C-h were treated with 100 μM SSZ for 3 h to assess the level of Cys-SSH with **Cy-Dise**. The cells in Fig. 2C-g showed an increase in the ratio image, while the cells in Fig. 2C-h displayed opposing results. The results indicated that the concentration of intracellular cystine had a direct impact on the generation of Cys-SSH. Flow cytometry analyses were performed for checking the fluorescence signal changes which were induced by Cys-SSH (Fig. 2C-i). All the above experiments indicated that the probe **Cy-Dise** could competently provide a ratio image for the detection of Cys-SSH in living cells.

### Visualization of Cys-SSH in peritoneal cavity of mice BALB/c

**Fig. 3:**
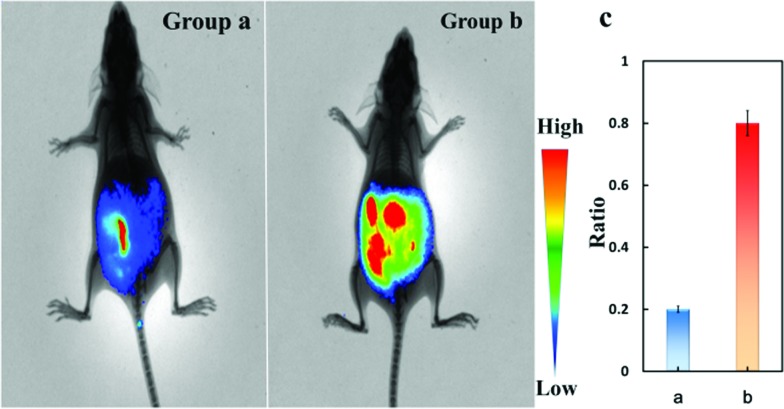
*In vivo* imaging of Cys-SSH in the peritoneal cavity of BALB/c mice. (a) Mice were injected in the i.p. cavity with Cys-SS-Cys (100 μM, 50 μL), PLP (1 μM, 50 μL) for 6 h. (b) Mice were injected in the i.p. cavity with Cys-SS-Cys (100 μM, 50 μL), PLP (1 μM, 50 μL) and Cu^2+^ (1 μM, 50 μL) for 6 h. Then the two groups were injected with **Cy-Dise** (1 μM, 50 μL, in 1 : 99 DMSO–saline, v/v) for 10 min. (c) Average values of (a) and (b). The experiments were repeated three times and the data are shown as the mean (±S.D.).

Near-infrared florescence can deeply penetrate into tissues and can avoid biological auto-fluorescence interference. Meanwhile, near-infrared florescence can reduce photodamage to biological samples. We then performed tests to explore the potential application of **Cy-Dise** as an *in vivo* imaging tool in mice. As shown in Fig. 3, the mice in group **a** were chosen as the control. The mice were injected with Cys-SS-Cys (100 μM, 50 μL) and PLP (1 μM, 50 μL) for 6 h. The mice in group **b** were injected with Cys-SS-Cys (100 μM, 50 μL), PLP (1 μM, 50 μL) and Cu^2+^ (1 μM, 50 μL) for 6 h. Then the two groups of BALB/c mice were given an intraperitoneal (i.p.) injection of **Cy-Dise** (1 μM, 50 μL, in 1 : 99 DMSO–saline, v/v) for 10 min before examining the changes to the ratio images. The *in vivo* assays were carried out on an *in vivo* imaging system (Bruker), and the images were reconstructed utilizing Image-Pro Plus software from two fluorescence collection windows, channel 1: *λ*_ex_ = 730 nm with filter 780 nm, and channel 2: *λ*_ex_ = 610 nm with filter 710 nm. Group **b** gave stronger ratio images than group **a** due to the high levels of Cys-SSH in mice (Fig. 3c). The results demonstrated that our probe **Cy-Dise** could achieve deep tissue imaging *in vivo.*

### Visualization of Cys-SSH in hepatic carcinoma models

**Fig. 4:**
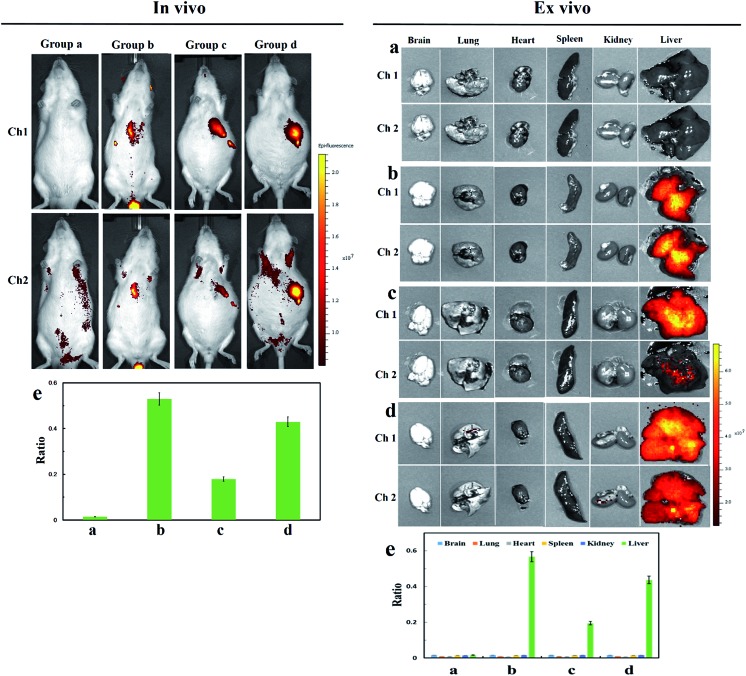
*In vivo* and *ex vivo* imaging of Cys-SSH by intravenous injection of **Cy-Dise** in SD rats and Walker-256 tumor SD rats. *In vivo*: (a) normal SD rats were injected with 50 μL solution (1 : 99 DMSO–saline, v/v) for 15 min. (b) Normal SD rats were injected with **Cy-Dise** (10 μM, 50 μL, in 1 : 99 DMSO–saline, v/v) for 15 min. (c) Walker-256 tumor SD rats were injected with **Cy-Dise** (10 μM, 50 μL, in 1 : 99 DMSO–saline, v/v) for 15 min. (d) Walker-256 tumor SD rats were injected with **Cy-Dise** (10 μM, 50 μL, in 1 : 99 DMSO–saline, v/v) for 45 min. (e) Average ratio intensity value in SD rats (a and b) and hepatic carcinoma SD rats (c and d). The experiments were repeated three times and the data are shown as the mean (±S.D.). *Ex vivo*: imaging of Cys-SSH in organs sacrificed from SD rats: group **a** – group **d**. (e) Ratio analysis for corresponding organs. The experiments were repeated three times and the data are shown as the mean (±S.D.).

ASGP-R specifically expresses on the plasma membrane of mammalian hepatocytes. We supposed that the termination of galactose would lead to **Cy-Dise** accumulating in the liver. The following *in vivo* imaging assays were performed on an *in vivo* imaging system (PerkinElmer). The fluorescence images were obtained from two fluorescence collection windows: channel 1: *λ*_ex_ = 730 nm with filter 780 nm, and channel 2: *λ*_ex_ = 610 nm with filter 710 nm. The normal Spraque-Dawley (SD) rats in group **a** were intravenously injected with 50 μL solution (1 : 99 DMSO–saline, v/v) as the control. The SD rats in group **b** were given an intravenous injection of **Cy-Dise** (10 μM, 50 μL, in 1 : 99 DMSO–saline, v/v) for 15 min. As shown in group **b** (Fig. 4 right plate), our probe **Cy-Dise** perfectly targeted the liver, indicating excellent liver positioning capability. The increasing fluorescence intensity in channel 2 evidenced the high level of Cys-SSH in the liver. The average value of the ratio image in group **b** was 0.53 (Fig. 4e left plate). We next confirmed that the rapid accumulation of **Cy-Dise** in the liver was attributed to the targeting unit: d-galactopyranoside. Immunohistochemical examinations proposed that the expression of ASGP-R is higher in tumorous liver than in non-tumorous liver.^47^ SD rats in groups **c** and **d** were Walker-256 tumor transplanted models. All the two groups were tail-vein injected **Cy-Dise** (10 μM, 50 μL, in 1 : 99 DMSO–saline, v/v) for 15 min and 45 min, respectively. It was incontestable that the carcinoma model (group **c**) exhibited stronger fluorescence in channel 1 compared to group **b**, which demonstrated that the overexpressed ASGP-R accelerated the accumulation of **Cy-Dise** in the liver. However, the low average value of the ratio image in group **c** indicated the relatively low level of Cys-SSH in tumor tissue (Fig. 4). The results were confirmed by extending the cumulative time to 45 min. As shown in Fig. 4 group **d**, the fluorescence intensities of the SD rats in channel 2 increased. The average value of the ratio image (group **d**) was 0.43 (Fig. 4e left plate). Carcinoma models were also evaluated and proved by H&E staining (Fig. S17†). *Ex vivo* imaging clearly indicated that the selective location of **Cy-Dise** was at the liver over other organs including the brain, lungs, heart, spleen, and kidney tissue (Fig. 4 right plate). The results were consistent with the experimental results of cells, which implied that the biosynthesis dysfunction on Cys-SSH generation might have a cause-and-effect relationship with carcinomas.

## Conclusion

In summary, we design and synthesize a ratiometric near-infrared fluorescent probe **Cy-Dise** for the qualitative and quantitative analyses of cysteine hydropersulfide in living cells and *in vivo*. The detection mechanism is based on a selenium–sulfur exchange reaction, and the fluorescence mechanism is manipulated *via* an efficient ICT process. The utility of the probe **Cy-Dise** for Cys-SSH ratio imaging has been fully demonstrated in terms of its outstanding sensitivity and selectivity. The ratio imaging analyses of HepG2 cells, HL-7702 cells, and primary hepatocytes confirm the quantitative and quantitative detection capabilities of **Cy-Dise** for Cys-SSH detection. The presence of the galactose group in **Cy-Dise** enables it to target the liver. The bioassays in BALB/c mice illustrate the application of ratio imaging in deep tissue. The examinations in Spraque-Dawley (SD) rats (normal and xenografts model of Walker-256 tumor) further exhibit the potential application of the probe for the detection of Cys-SSH in the liver. We anticipate that **Cy-Dise** has promising applications in the investigation of Cys-SSH related roles in physiological and pathological processes.

## Supplementary Material

Supplementary movie

Supplementary movie

Supplementary information
