# Supplementary material for: Quantification of cysteine hydropersulfide with a ratiometric near-infrared fluorescent probe based on selenium–sulfur exchange reaction
Source: Chem Sci. 2016 Apr 11;7(8):5098–107. doi: 10.1039/c6sc00838k (PMC6020118; doi:10.1039/c6sc00838k)
Supplement: Supplementary file 3 [file SC-007-C6SC00838K-s003.pdf]

Electronic Supplementary Material (ESI) for Chemical Science.
This journal is © The Royal Society of Chemistry 2016

Supporting Information

Quantification of cysteine hydropersulfide with a ratiometric near-infrared fluorescent probe based on selenium-sulfur exchange reaction

Xiaoyue Han,[ab†] Fabiao Yu,[a†] Xinyu Song,[a] Lingxin Chen[a*]

[a]Key Laboratory of Coastal Environmental Processes and Ecological Remediation, Yantai Institute of Coastal Zone Research, Chinese Academy of Sciences, Yantai 264003, China.

[b]University of Chinese Academy of Sciences, Beijing 100049, China

†The authors contributed equally.

Contents:

1. General Methods
2. Synthesis and Characterization of Compounds
3. Absorption Spectra of **Cy-Dise** upon Treatment of Cys-SSH
4. $F_{797\ nm}$ and $F_{749\ nm}$ upon Treatment of Cys-SSH
5. Effect of pH Values to **Cy-Dise** and Cy
6. Selectivity of **Cy-Dise** towards Various Reactive Substances
7. Reaction Kinetics of **Cy-Dise** towards Cys-SSH
8. Reaction Mechanism Discussion between Probe **Cy-Dise** and Cys-SSH
9. MTT Assay for **Cy-Dise**
10. Sublocation in Cells
11. Plots of Relative Fluorescence Intensities of **Cy-Dise** against Time for Fig. 4A and Fig. 4B
12. Flow Cytometry Analysis for Fig. 4A and Fig. 4B
13. Flow Cytometry Analysis for Fig. 4C
14. Bright-field Images of Fig. 4B
15. Bright-field Images of Fig. 4C
16. Time-dependent Ratio Images of Cys-SSH Generation in NEM Treated Primary Hepatocytes
17. LC-MS/MS Analysis for Cys-SSH in Cell Lysates
18. H&E Staining of the Liver of Normal Rat and Hepatic Carcinoma Rat
19. *In vivo* and *ex vivo* imaging of Cys-SSH in Walker-256 tumor SD rats by control probe.
20. Supplementary Movie 1
21. Supplementary Movie 2
22. ^{1}H NMR, ^{13}C NMR and HRMS of Probe **Cy-Dise**
23. References

1. General Methods

Materials: The solution of the probe **Cy-Dise** (1 mM) could be dissolved in dimethyl sulfoxide (DMSO) and maintained in refrigerator at 4 °C. The purity of probe was tested on a Shimadzu LC-20AT HPLC system equipped with fluorescence and UV-vis absorption detectors. When it was used for imaging, the purity of our probe was greater than 99.89%. Stock solutions of cysteine (Cys), homocysteine (HCy), glutathione (GSH), cysteine (Cys-SS-Cys), oxidized glutathione (GSSG), $S_2O_3^{2-}$, HSO_3^-, metallotionein, lipoic acid, ascorbic acid, tocopherol were prepared to desired concentrations when needed. DL-propargylglycine (PAG) and hydroxylamine (HA) were prepared in saline (1 mM). The $CuCl_2$ and pyridoxal phosphate were dissolved in saline. In 0.2 mL 0.3 mol/L HCl, 25 µmol Cystine was dissolved, and then water was added to dilute the total volume to be 1.5 mL.[1] Stock solutions of 25 mM sulfasalazine (SSZ) was prepared by dissolving SSZ in 50 mM $NaHCO_3$ at 37 °C. Following filter sterilization (through a 0.220-µm filter), stored at -20 °C.[2] Methyl linoleate (MeLH) and 2,2'-azobis-(2,4-dimethyl)valeronitrile (AMVN) were used to produce MeLOOH.[3] OCl^- was standardized at pH 12 ($\varepsilon_{292\ nm}$ = 350 $M^{-1}cm^{-1}$).[4] H_2O_2 was determined at 240 nm ($\varepsilon_{240\ nm}$ = 43.6 $M^{-1}cm^{-1}$). $O_2^{\cdot -}$ was created by the enzymatic reaction of xanthine/xanthineoxidase (XA/XO; 6.0 µM/3 mU) at 25 °C for 5 min.[5] ·OH was generated by Fenton reaction between Fe^{II}(EDTA) and H_2O_2 quantitively, and Fe^{II}(EDTA) concentrations represented ·OH concentrations. *Tert*-butylhydroperoxide (*t*-BuOOH) and cumene hydroperoxide (CuOOH) could also be used to induce ROS in biological systems.[6] The $ONOO^-$ source was the donor 3-Morpholinosydnonimine hydrochloride (SIN-1, 200 µmol/mL).[7] NO was generated in form of 3-(Aminopropyl)-1-hydroxy-3-isopropyl-2-oxo-1-triazene (NOC-5, 100 µM/mL).[8] NO_2^- was generated from $NaNO_2$. S-nitrosoglutathione (GSNO) was synthesized from GSH, according to the published procedure.[9] S-nitrosoacetyl-penicillamine (SNAP) was synthesized from l-penicillamine and methoxycarbonylsulfenyl chloride.[10] Protein sulfenic acids (AhpC-SOH) was prepared according to the published procedure.[11] Glutathione persulfide (GSSH) was synthesized from glutathione (oxidized form) and freshly sodium sulfide (Na_2S) in Tris-HCl buffer (pH 7.4) at 37 °C for 15 min.[12] Papain persulfide was generated from papain, 5′-dithiobis-2-nitrobenzoic acid and Na_2S at 25 °C for 15 min and then purified by PD-10 column according to the published procedure.[12] Gpx3 persulfide was synthesized from Gpx3, 5′-dithiobis-2-nitrobenzoic acid and freshly Na_2S according to the published procedure.[12,13] Human serum albumin persulfide (HSA-SSH) was synthesized from human serum albumin (HSA) and HS_x^- according to the published procedure.[14] The original protein were purchased from Sigma-Aldrich or Fisher. Na_2S_4 was synthesized from sulfur and sodium according to the published procedure.[15] Cys-polysulfide was prepared using reported procedures.[16] The stock solutions were diluted to desired concentrations when needed. HEPES (4-(2-Hydroxyethyl)-1-piperazineethanesulfonic acid) was obtained from Aladdin and 3-(4,5-Dimethylthiazol-2-yl)-2,5-diphenyltetrazolium bromide (MTT) was purchased from Sigma-Aldrich. All other chemicals were from commercial sources and of analytical reagent grade, unless indicated otherwise. Ultrapure water was used throughout.

Instruments: Absorption spectra were obtained on Lambda 35 UV-visible spectrophotometer (PerkinElmer). Fluorescence spectra were measured by FluoroMax-4 Spectrofluorometer with a Xenon lamp and 1.0-cm quartz cells. ^1H and ^{13}C NMR spectra were taken on a Bruker spectrometer. High-resolution mass spectra were carried on LCQ Fleet LC-MS System (Thermo Fisher Scientific). The fluorescence images of cells were acquired using a LTE confocal laser scanning microscope (Olympus FV1000 confocal laser-scanning microscope) with an objective lens (× 40/60). BALB/c mice images were collected by Bruker In-vivo Imaging System and Walker256 tumor cells induced hepatic carcinoma SD rats images were captured by Xenogen IVIS Spectrum Pre-clinical In Vivo Imaging System. Walker-256 tumor-bearing rat liver pathological sections were imaged by Motic BA400 Biological Research Compound Infinity Binocular Microscope.

Absorption and Fluorescence Analysis: Absorption spectra were obtained with 1.0-cm glass cells. Fluorescence emission spectra were obtained with a Xenon lamp and 1.0-cm quartz cells. The fluorescence intensity were measured at $\lambda_{ex/em}$ = 730/780-840 nm and at $\lambda_{ex/em}$ = 614/650-850 nm, respectively. The **Cy-Dise** (0.10 mL, 1.0 mM) was added to a 10.0-mL color comparison tube. After dilution to 10 μM with 10 mM HEPES buffers, analytes were added. The mixtures were equilibrated 2 min before measurement.

Cell Culture: HepG2 cells and HL-7702 cells were purchased from the Committee on Type Culture Collection of Chinese Academy of Sciences (Shanghai, China). HepG2 cells were incubated in DMEM (Dulbecco's Modified Eagle Medium) supplemented with 10% fetal bovine serum (FBS). HL-7702 cells were incubated in RPMI (Roswell Park Memorial Institute) 1640 Medium supplemented with 10% (FBS) at 37 °C under a humidified atmosphere containing 5% CO_2. The cells were passaged by scraping and seeding on 20 mm Petri-dishes according to the instructions from the manufacturer.

Isolation and Culture of Hepatocytes: The primary hepatocytes were acquired from 6- to 8-weeks-old female BALB/c mice. Hepatocytes were isolated by in situ collagenase perfusion method.[17] Primarily, the liver was perfused by perfusion buffer and collagenase buffer through a needle aligned along the inferior vena cava. Then the liver was dissected, suspended in Hanks solution and filtered through cheesecloth and 100 mm nylon membrane to remove connective tissue debris and cell clumps. Hepatocytes were purified by centrifugation (42 *g* force, 2 min) for three times at 4 °C and then by a density gradient centrifugation (42 *g* force, 2 min) at 4 °C using 45% Percoll solution. The isolated hepatocytes (25,000 cells/cm^2) were allowed to adhere onto 20 mm Petri-dishes in Williams' Medium E, pH 7.3, containing 10 mM $NaHCO_3$, 50 mg/ml penicillin and streptomycin, and 100 mg/mL neomycin (WE medium) under humidified atmosphere of 5% CO_2 and 95% air for 3 h at 37 °C. Fresh WE medium was added to remove non-adherent hepatocytes. And the adherent cells were cultured for an indicated time.[18]

Cell Imaging: Florescent images were acquired on Olympus FV1000 confocal laser-scanning microscope with an objective lens (× 40/60). The excitation wavelength was chosen as described in paper. Cells were plated on Petri-dishes (Φ = 20 mm) and allowed to adhere for 24 hours before imaging. The probe was added to the culture plates which were filled with 1 mL fresh complete medium.

Flow Cytometry Analysis: The cells were cultured at 2.0 × 10^5 cells/well in 6-well plates, and then treated with probes as described in the paper. After harvest, cells were washed and suspended in fresh complete medium and analyzed by flow cytometry. Excitation wavelength was 633 nm. The collected wavelengths were 750 - 810 nm (channel 1) and 650 - 670 nm (channel 2).

LC-MS/MS Analysis for Cys-SSH in Cell Lysates: Analytical LC-MS data for Cys-SSH were acquired by using LCQ Fleet LC-MS System (Thermo Fisher Scientific) or TSQ Quantum Access MAX LC-MS System (Thermo Fisher Scientific). Cys, P-NONOate and NaHS were used to synthesize Cys-SSH, which is tagged by Br-bimane to form Cys-SS-bimane.[19] CysS-$(S)_n$-bimane adducts thus formed were separated on the basis of sulfur number by RP-HPLC. Preparative HPLC was performed with the UV detector set at 210 nm. The cultured HepG2, HL-7702 cells and primary hepatocytes were washed with PBS buffer twice. After centrifugation and removal of supernatant liquid, the resulting cells were stored at -80°C for 5 min. After thawing to room temperature, the cells were lysed by hyperacoustic in 1 mL lysis buffer (PBS with 0.1% Triton X-100, pH 7.4) and the supernatant liquid was collected after centrifugation (10,000 × *g*, 10 min, 4 °C). Then cell lysis samples (10 μL) were incubated with Br-bimane (5 mM, 50 μL) at 37 °C for 15 min to use.

Analytical LC-MS/MS data for Cys-SSH in the cell lysis samples were acquired by using TSQ Quantum Access MAX LC-MS System (Thermo Fisher Scientific), with a linear 5% – 90% methanol gradient for 14 min in 0.1% formic acid at 40 °C. A total flow rate of 0.2 mL/min and an injection volume of 20 µL were used. Ionization was achieved by using electrospray in the positive mode with a 3,500 V spray voltage. Nitrogen was the nebulizer gas, with the nebulizer pressure set at 50 psi. The desolvation gas (nitrogen) was heated to 350 °C and was delivered at the flow rate of 10 L/min. Collision-induced dissociation (CID) was achieved by using high-purity nitrogen as the collision gas at a pressure of 0.5 MPa. Mass spectra were recorded in the positive ion mode.

Animals: 5-weeks-old female BALB/c mice weighing 135 to 140 g, 2-week-old female SD rats weighing 60 to 80 g and 8-week-old female SD rats weighting 150 to 180 g were obtained from Binzhou Medical University. The animals were housed in cages and fed a standard laboratory diet and tap water *ad libitum*. Surgical procedures were carried out under anesthesia with ketamine hydrochloride (100 mg/kg, ip). All experiments were performed in accordance with the guidelines established by the Committee of Animal Research Policy of Binzhou Medical University.

Establish of the Walker-256 transplanted hepatoma SD Rats: Walker-256 carcinoma cells were purchased from Cell Preserve Center (Wuhan University, Wuhan, China). 2-week-old rats were subcutaneously inoculated 0.5 mL cell suspension (2×10^7/mL). After a week, the 2-week-old subcutaneous tumor was sharply minced into small cubes about 1 mm × 1 mm × 1 mm (0.5 mg), corresponding to $1 \times l0^5$ tumor cells, and was prepared as tumor inocula. After depilation and disinfection, the 8-week-old rats underwent a small subxiphoid midline abdominal incision. The left lateral lobe of the liver was gently retracted out of the abdominal cavity. A small superficial incision on the liver was made using the tip of a surgical blade with the knife at a 30° - 45° angle to the liver surface. A small piece of solidified gelfoam was inserted into the incision. Approximately 1 - 2 min later, the bleeding had stopped and the gelfoam was removed. And a tumor fragment was gently placed into the pocket with an angled forceps so that the tumor was completely buried in hepatic parenchyma. The hepatic lobe was gently returned to the peritoneal cavity and the abdominal wall was closed.[20]

H&E staining: Livers from normal rats and hepatic carcinoma rats were excised and fixed in 10% formaldehyde and embedded in paraffin. Then the treated livers were prepared to frozen sections and stained with hematoxylin and eosin (H&E) to confirm tumor histology.

Experiments of BALB/c Mice: A Bruker In-vivo Imaging System was used for bio-imaging in this group of animal experiments. The excitation and emission wavelength were chosen as described in paper. Mice were anesthetized prior to injection and during imaging via inhalation of isoflurane.

Experiments of Hepatic Carcinoma Models: Hepatic carcinoma models were built and then Xenogen IVIS Spectrum Pre-clinical In Vivo Imaging System was used for bio-imaging. Rats were anesthetized prior to injection and during imaging via inhalation of isoflurane. After *in vivo* imaging, the organs (such as brain, lung, heart, spleen, kidney and liver) were excised to perform *ex vivo* imaging with Xenogen IVIS Spectrum Pre-clinical In Vivo Imaging System. The excitation and emission wavelength were chosen as described in paper.

2. Synthesis and Characterization of Compounds

Synthesis of compound 8. Sodium borohydride (2.27 g, 0.06 mol) was resolved in 50 mL ultrapure water under Ar atmosphere. Selenium (2.4 g, 0.03 mol) was added to the mixture in batches at 25 °C with magnetic stirring. After

the mixture changed to milk white, another dosage of selenium (2.4 g, 0.03 mol) was added. After refluxed for 3 h, the mixture was cooled down to room temperature. 2-bromoethanol (7.87 g, 0.03 mol) in 100 mL THF was added into the mixture dropwisely.[21] Then the reaction was refluxed for more 12 h, and the obtained solution was extracted with CH_2Cl_2 (20 mL × 3). After dried with anhydrous Na_2SO_4, the organic phase was concentrated. The residue was purified by silica column chromatography (200 - 300 mesh) with CH_2Cl_2 as eluent. The product was obtained as yellow oil (1.68 g, yield: 24%). ^{1}H NMR (500 MHz, $CDCl_3$-D_1) δ (ppm): 4.01-3.98 (t, 1H), 3.93-3.91 (t, 4H), 3.31-3.28 (t, 1H), 3.12-3.09 (t, 4H). ^{13}C NMR (125 MHz, DMSO-D_6) δ (ppm): 61.75, 32.64.

Synthesis of compound 6. Sodium hydride (60% in paraffin, 0.60 g, 15 mmol) was resolved in 15 mL anhydrous DMF with a 250 mL round-bottom flask, then stirred for 20 min. 4-(4-hydroxyphenyl)Cyclohexanone (2.85 g, 15 mmol) which was resolved in 15 mL anhydrous DMF was added slowly at 0 °C. Propargyl bromide (1.19 g, 10 mmol) in 100 mL anhydrous DMF was dropwisely added into above solution within 60 min. The reacting mixture was stirred overnight at 25 °C, and the reaction was quenched by adding water.[22] After extracted with CH_2Cl_2 (20 mL × 3), the organic layer was washed with saturated NaCl aqueous (20 mL × 3), and then dried over by anhydrous Na_2SO_4. After concentrated, the residue was purified by silica column chromatography (200 - 300 mesh) with CH_2Cl_2 as eluent to give a white solid (1.894 g, yield: 55%).^{1}H NMR (500 MHz, $CDCl_3$-D_1) δ (ppm): 7.19-7.17 (d, 2H), 6.95-6.93 (d, 2H), 4.67-4.67 (d, 2H), 3.01-2.96 (m, 1H), 2.53-2.48 (m, 5H), 2.19-2.18 (m, 2H), 1.92-1.88 (m, 2H). ^{13}C NMR (125 MHz, $CDCl_3$-D_1) δ (ppm): 211.10, 156.05, 137.74, 127.50, 114.82, 78.55, 75.43 55.70, 41.77, 41.24, 34.02. LC-MS (ESI$^-$): m/z $C_{15}H_{16}O_2$ calcd. 228.1150, found [M-H]$^-$ 227.1085.

Synthesis of compound 7. 2, 3, 3-trimethyl-3H-indolenine (24.8 g, 156 mmol) and iodoethane (30 g, 192 mmol) were added in a 250 mL round flask with 50 mL acetonitrile.[23] The mixture was refluxed for 12 h under argon atmosphere. After cooled down to room temperature, the precipitate was filtered through a Buchner funnel. The pink solid was washed by diethyl ether and then afforded **7** (39.1 g, yield: 80%). ^{1}H NMR (500 MHz, DMSO-D_6) δ (ppm): 8.05-8.03 (t, 1H), 7.92-7.89 (t, 1H), 7.67-7.62 (m, 2H), 4.58-4.53 (m, 2H), 2.92 (s, 3H), 1.58 (s, 6H), 1.50-1.46 (t, 3H). ^{13}C NMR (125 MHz, DMSO-D_6) δ (ppm): 196.55, 142.45, 141.19, 129.44, 124.12, 115.88, 54.66, 43.80, 40.17, 22.45, 14.88, 13.34 LC-MS (ESI$^+$): m/z $C_{13}H_{18}N^+$ calcd. 188.1434, found [M$^+$] 188.1434.

Synthesis of compound 5. A mixture of 15 mL anhydrous DMF and 15 mL anhydrous CH_2Cl_2 was placed in a 250 mL round-bottom flask at -10 °C stirring for 20 min. Phosphorus oxychloride (16.45 g, 106 mmol) which was dissolved in 15 mL of anhydrous CH_2Cl_2 was dropwisely added into above solution. After compound **6** (4.5 g, 20 mmol) was added into the mixture in batches, the solution changed from colorless to yellow immediately. The solution was refluxed for 3 h, then the mixture was poured into 200 g ice, and allowed to stand overnight. The yellow precipitation was collected through a Buchner funnel. After dried with phosphorus pentoxide in desiccator, compound **5** could be preserve in petroleum ether (60 - 90 °C) at 0 °C for more than three weeks (2.58 g, yield: 43%). 9.80 (s, 1H), 7.40-7.37 (m, 2H), 6.88-6.86 (m, 2H), 6.40 (s, 1H), 5.12 (s, 1H), 4.92 (s, 2H), 3.36 (s, 1H), 2.95 (m, 1H), 2.20-2.15 (m, 4H). ^{13}C NMR (DMSO-d^6, 125 MHz) δ (ppm): 191.7, 162.7, 155.6, 142.5, 134.7, 129.1, 129.0, 120.9, 117.8, 79.5, 76.8, 58.1, 38.9, 30.7. LC-MS (ESI$^-$): m/z $C_{17}H_{15}ClO_3$ calcd. 302.0710, found [M-H]$^-$ 301.0635.

Synthesis of compound 4. Compound **5** (1.0 g, 2 mmol) and **7** (0.752 g, 4 mmol) were resolved in 150 mL mix solution of *n*-butyl alcohol and benzene (7:3 v/v), refluxed for 3 h.[24] After evaporated with rotary evaporator, the crude product was purified on silica gel chromatography (200 - 300 mesh) with gradient eluent of CH_2Cl_2 and CH_3OH (100:0 to 90:10 v/v) to afford compound **4** as green solid (0.89 g, yield 70%).^{1}H NMR (500 MHz, $CDCl_3$-

D_1) δ (ppm): 8.43-8.40 (d, 2H), 7.42-7.38 (m, 6H), 7.28-7.21 (m, 5H), 7.07-7.06 (d, 2H), 6.10-6.07 (d, 2H), 4.73-4.73 (d, 2H), 4.19-4.09 (m, 6H), 3.06-3.00 (m, 3H), 2.74-2.69 (q, 2H), 2.56-2.55 (t, 1H), 2.04 (s, 3H), 1.74 (s, 3H), 1.43-1.40 (t, 6H), 1.27-1.24 (t,3H). ^{13}C NMR (125 MHz, $CDCl_3$-D_1) δ (ppm): 172.19, 156.57, 150.06, 144.48, 141.53, 141.07, 137.11, 128.89, 128.33, 126.32, 125.49, 122.27, 115.18, 110.88, 100.57, 78.58, 75.53, 60.28, 55.83, 49.44, 39.95, 37.95, 34.04, 28.01, 27.97, 20.95, 14.10, 12.42. LC-MS (ESI+): m/z $C_{43}H_{46}ClN_2O^+$ calcd. 641.3293, found [M^+] 641.3293.

Synthesis of compound 3. Compound **4** (1.278 g, 2 mmol) and methylamine hydrochloride (1.332 g, 20 mmol) triethylamine (2.02 g, 2.8 mL, 20 mmol) were dissolved in 50 mL anhydrous DMF.[25] The mixture was stirred for 24 h at 40 °C under Ar atmosphere. The crude product was purified on silica gel chromatography (200 - 300 mesh) with gradient eluent of CH_2Cl_2 and CH_3OH (100:0 to 90:10 v/v) to afford compound **3** as deep blue solid (1.10 g , yield 87%). ^{1}H NMR (500 MHz, $CDCl_3$-D_1) δ (ppm): 7.36-7.34 (d, 2H), 7.30-7.26 (m, 4H), 7.08-7.04 (t.2H), 7.02-7.00 (d, 2H), 6.96-6.94 (d, 2H), 4.89 (s, 4H), 4.69-4.69 (d, 2H), 4.12-4.07 (q, 1H), 3.92-3.90 (q, 4H), 3.10-3.05 (m, 1H), 2.94-2.90 (q, 2H), 2.82-2.81 (t, 1H), 2.66-2.61 (q, 2H), 2.01 (s, 1H), 1.66-1.61 (d, 12H), 1.27-1.22 (m,8H). ^{13}C NMR (125 MHz, $CDCl_3$-D_1) δ (ppm): 173.12, 171.52, 168.42, 157.97, 144.15, 141.53, 139.47, 139.42, 129.53, 129.28, 126.63, 123.91, 123.21, 119.81, 116.17, 109.78, 94.78, 80.10, 76.74, 61.68, 56.72, 49.33, 48.99, 39.83, 37.83, 34.56, 29.20, 29.09, 21.02, 14.62, 11.79. LC-MS (ESI+): m/z $C_{44}H_{50}N_3O^+$ calcd. 636.3948, found [M^+] 636.3948.

Synthesis of compound 2. Aqueous solutions of sodium ascorbate (0.01 M, 1 mL) and $CuSO_4 \cdot 5H_2O$ (0.01 M, 1 mL) were mixed to produce an orange suspension containing the copper (I) catalytic species under Ar atmosphere.[26] Compound **3** (63.7 mg, 0.10 mmol), acetyl-D-galactopyranoside (55.5 mg, 0.11 mmol) and DIPEA (1.5 g, 0.01 mmol) were subsequently added into 6.0 mL methanol. The mixture was further stirred for 24 h at 25 °C under Ar atmosphere. The solvent was removed in vacuum and the obtained solid residue was purified through a silica gel chromatography (200 - 300 mesh) with gradient eluent CH_2Cl_2 and CH_3OH (100:0 to 85:15 v/v). Compound **2** was afforded as blue solid (22.8 mg , yield 20%).^{1}H NMR (500 MHz, $CDCl_3$-D_1) δ (ppm): 8.27-8.24 (d, 1H) 7.92-7.91 (t, 1H), 7.37 (m, 1H), 7.21-7.08 (m, 5H), 6.94-6.87 (m, 3H), 6.87-6.85 (m, 1H), 6.70-6.69 (m, 1H), 5.46-5.44 (m, 2H), 5.31 (s, 1H), 5.23 (m, 2H), 4.59 (m, 1H), 4.33-4.30 (m, 9H), 4.18-4.15 (m, 4H), 3.70-3.63 (m, 8H), 3.00 - 2.97 (m, 3H), 2.63 (t, 1H), 2.34 (m, 2H), 2.23 (m, 2H), 2.15 (m, 2H), 2.06-2.00 (m, 13H), 1.67 (s, 6H), 1.31-1.11 (m, 12H). ^{13}C NMR (125 MHz, $CDCl_3$-D_1) δ (ppm): 186.72, 185.81 172.79, 170.39, 170.23, 170.15, 169.44, 142.54, 139.89, 139.67, 128.15, 127.70, 124.02, 122.49, 122.43, 122.24, 121.94, 121.83, 114.91, 108.18, 108.02, 101.38, 98.50, 70.88, 70.70, 70.59, 70.40, 70.24, 69.46, 69.17, 68.84, 67.04, 62.11, 61.23, 53.44, 50.36, 47.59, 46.76, 37.63, 37.02, 33.92, 27.22, 22.69, 20.78, 20.69, 20.66, 20.59, 14.12, 11.24, 11.11. LC-MS (ESI$^+$): m/z $C_{64}H_{81}N_6O_{13}^+$ calcd. 1141.5856, found [M^+] 1141.5852.

Synthesis of compound 1 (Cy-Dise). Compound **2** (0.12 g, 0.1 mmol), triphosgene (0.09 g, 0.3 mmol) was dissolved in 50 mL anhydrous CH_2Cl_2 under Ar atmosphere.[27] The mixture was suspended at 0 °C, then 1 mL DIPEA was added. The reaction was lasted for 30 min, and the color of the solution changed into green from blue. After removed solvent in vacuum, the obtained residue was dissolved in 50 mL anhydrous CH_2Cl_2, and added DIPEA (1 mL) and DMAP (20 mg). Compound **8** (0.050 g, 0.2 mmol) in CH_2Cl_2 (2 mL) was added into above mixture, then the reaction mixture was stirred at 25°C, TLC monitored the reaction until the starting material was completely consumed. The obtained solid residue was purified through a silica gel chromatography (200 - 300 mesh) with gradient eluent of CH_2Cl_2 and CH_3OH (100:0 to 85:15 v/v). The solvent was removed in vacuum, and the obtained solid was dissolved in CH_2Cl_2, filtered and evaporated to give a green solid (115 mg, yield 87 %). LC-

MS (ESI^+): m/z $C_{69}H_{89}N_6O_{16}Se_2^+$ calcd. 1417.4660, found [M^+] 1417.4663. The green solid (85.0 mg, 0.06 mmol) and sodium methoxide (1.2 mL from a 0.5 M solution in MeOH) was added in anhydrous MeOH (5 mL). The reaction mixture was stirred at 25 °C for 1 h. After neutralized with diluted hydrochloric acid (5%), the solution was extracted with CH_2Cl_2 (3 × 50 mL). The organic layer was dried with anhydrous Na_2SO_4. After concentrated under vacuum, the crude solid was purified by column chromatography (200 - 300 mesh) using CH_2Cl_2: CH_3OH (8:1 v/v) as eluent to obtain **Cy-Dise** as green solid (52 mg, 61 %). ^1H NMR (500 MHz, $CDCl_3$-D_1) δ (ppm): 7.92-7.90 (m, 1H), 7.63-7.61 (m, 1H), 7.34-7.28 (m, 6H), 7.23-7.22 (m, 1H), 7.13-7.10 (m, 2H), 7.05-7.04 (m, 1H), 6.06-6.00 (d, 1H), 5.32 (m, 2H), 5.18 (m, 2H), 5.12-5.11 (s, 3H) 4.97-4.95 (m, 2H), 4.38-4.36 (m, 3H), 4.21 (s, 1H), 4.10-4.08 (m, 3H), 4.00 (s, 1H), 3.69-3.51 (m, 22H), 3.10-3.02 (m, 3H), 2.09-2.07 (m, 4H), 1.99 (s, 6H), 1.19-1.08 (m, 16H). ^{13}C NMR (125 MHz, $CDCl_3$-D_1) δ (ppm): 177.01, 174.47, 157.28, 155.65, 153.22, 142.45, 141.51, 140.98, 140.26, 136.87, 129.85, 129.06, 128.31, 126.19, 126.03, 125.62, 124.49, 122.38, 115.25, 110.82, 110.77, 101.23, 99.01, 72.09, 70.87, 70.48, 69.96, 69.85, 68.82, 67.11, 64.60, 61.87, 61.27, 52.45, 50.63, 50.38, 49.32, 47.56, 36.78, 34.23, 33.05, 32.56, 29.04, 28.44, 28.02, 15.93, 13.62. LC-MS (ESI^+): m/z $C_{61}H_{81}N_6O_{12}Se_2^+$ calcd. 1249.4237, found [M^+] 1249.4232.

3. Absorption Spectra of Cy-Dise upon Treatment of Cys-SSH

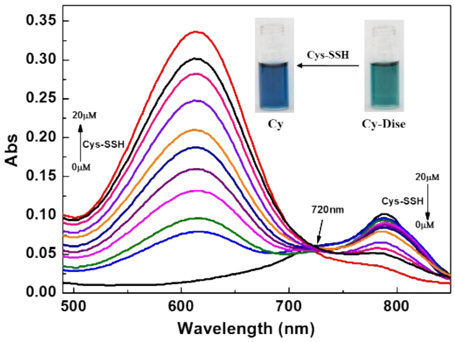

Fig. S1 Dose-dependent absorbance spectra of Cy-Dise towards Cys-SSH. **Cy-Dise** (10 μM) was incubated with increasing concentration of Cys-SSH (0 - 20 μM) at 37 °C in HEPES (pH 7.4, 10 mM).

4. $F_{797\ nm}$ and $F_{749\ nm}$ upon Treatment of Cys-SSH

The fluorescent intensity at 797 nm ($F_{797\ nm}$) and the fluorescent intensity at 749 nm ($F_{749\ nm}$) have linear relationship with the treatment of Cys-SSH (0 - 20 μM). $F_{797\ nm} = -7.26\times10^4$ [Cys-SSH] (μM) + 1.65×10^6 (r = 0.9989) and $F_{749\ nm} = 2.99\times10^4$ [Cys-SSH] (μM) + 4.11×10^4 (r = 0.9975), respectively.

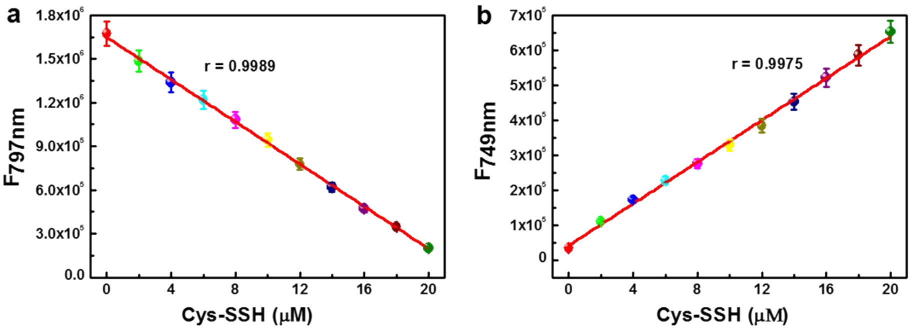

Fig. S2 $F_{797\ nm}$ (a) and $F_{749\ nm}$ (b) upon treatment of Cys-SSH (0 - 20 μM) at 37 °C in HEPES (pH 7.4, 10 mM). $F_{797\ nm}$: λ_{ex} = 730 nm, λ_{em} = 797 nm; $F_{749\ nm}$: λ_{ex} = 614 nm, λ_{em} = 749 nm.

5. Effect of pH Values to Cy-Dise and Cy

Standard fluorescence pH titrations were performed in 10 mM HEPES solution at a concentration of 10 μM (**Cy-Dise** and **Cy**). As shown in Fig. S3, the pH of the mediums hardly effected on fluorescence intensity within the range from 3.0 to 10.0. On this basis, we suggested that **Cy-Dise** would work well under physiological conditions (pH = 7.4).

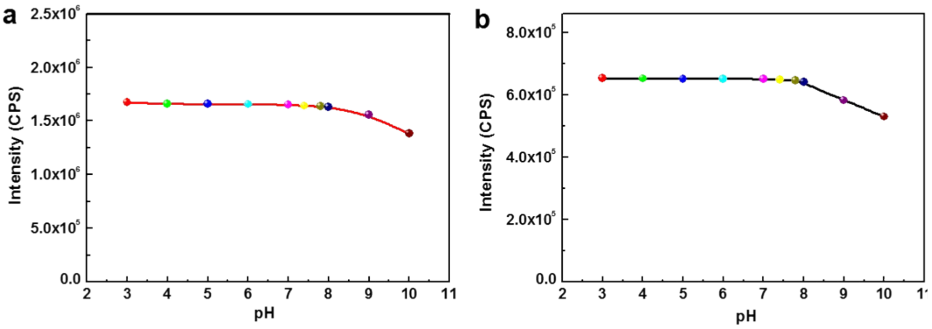

Fig. S3 Effect of pH values. a) The fluorescence emission changes at 797 nm with the pH titration curve of **Cy-Dise** (10 μM). **b)** The fluorescence emission changes at 749 nm with the pH titration curve of **Cy** (10 μM). pH: 3.0, 4.0, 5.0, 6.0, 7.0, 7.4, 7.8, 8.0, 9.0, 10.0 (10 mM HEPES buffer).

6. Selectivity of Cy-Dise toward Various Reactive Substances

UV absorbance spectra of probe with various analytes were carried out to further verify the selectivity of the probe. Cy-Dise (10 μM) was treated with various analytes in HEPES buffer (10 mM, pH 7.4) at 37 °C. The emission and UV absorbance spectra were recorded after 60 min. As shown in Fig.S4, reactive oxygen species, reactive nitrogen species, anions and metal ions, as wells as reactive sulfur species and reactive selenium species could not induce interference.

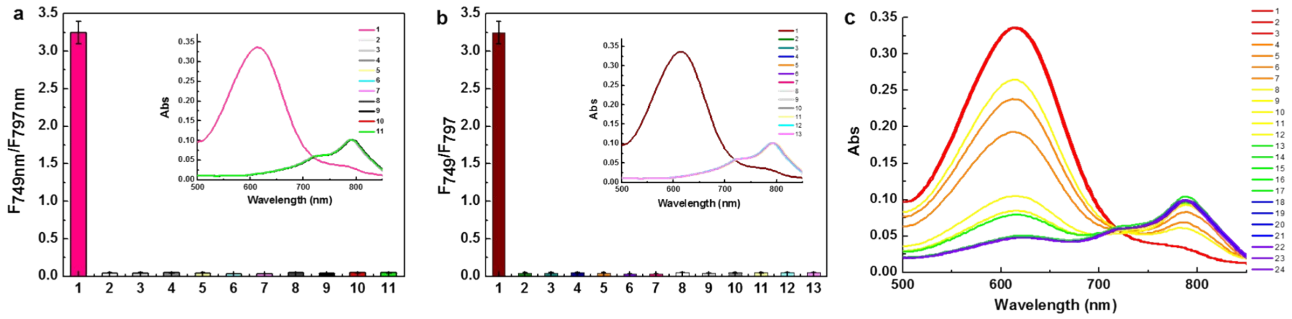

Fig. S4 Fluorescent ratio ($F_{749\ nm}/F_{797\ nm}$) response and absorbance spectra of Cy-Dise (10 μM) to reactive oxygen species, reactive nitrogen species, anions and metal ions, as wells as reactive sulfur species and reactive selenium species in HEPES buffer (pH 7.4, 10 mM) at 37 °C. a) 1, 20 μM Cys-SSH; 2, 200 μM NO_2^-; 3, 200 μM $ONOO^-$; 4, 200 μM NO; 5, 200 μM t-BuOOH; 6, 200 μM H_2O_2; 7, 200 μM $O_2^{\cdot -}$; 8, 200 μM MeLOOH; 9, 200 μM ClO^-; 10, 200 μM CuOOH, 11, 20 μM GSNO. b) 1, 20 μM Cys-SSH; 2, 1 mM K^+; 3, 1 mM Na^+; 4, 1 mM Ca^{2+}; 5, 1 mM Mg^{2+}; 6, 1 mM Zn^{2+}; 7, 1 mM Cu^{2+}; 8, 1 mM Cl^-; 9, 1 mM Br^-; 10, 1 mM F^-; 11, 1 mM CO_3^{2-} ; 12, 1 mM $H_2PO_4^-$; 13, 1 mM Critrate. c) 1, 20 μM Cys-SSH; 2, 20 μM GSSH; 3, 20 μM persulfide P; 4, 60 μM human serum albumin persulfide; 5, 60 μM Papain persulfide; 6, 60 μM Gpx3 persulfide; 7, 100 μM NaHS; 8, 100 μM Cys; 9, 20 μM cysteine methyl ester; 10, 40 μM Cys-polysulfide; 11, 20 μM Na_2S_4; 12, 20 μM Na_2S_4 + 40 μM Cys-polysulfide; 13, 100 μM Hcys; 14, 1 mM GSH; 15, 50 μM Cys-SS-Cys; 16, 50 μM $S_2O_3^{2-}$; 17, 50 μM HSO_3^-; 18, 50 μM AhpC-SOH; 19, 50 μM GSSG; 20, 50 μM lipoic acid; 21, 100 μM ascorbic acid; 22, 50 μM tocopherol; 23, 50 μM metallotionein; 24, 50 μM selenosysteine. $F_{749\ nm}$: λ_{ex} = 614 nm, λ_{em} = 749 nm; $F_{797\ nm}$: λ_{ex} = 730 nm, λ_{em} = 797 nm. The emission and UV absorbance spectra were recorded after 60 min.

7. Reaction Kinetics of Cy-Dise towards Cys-SSH

Considering the rapid metabolism unstable nature of Cys-SSH, the reaction kinetics of **Cy-Dise** towards Cys-SSH was performed to inspect whether the probe can fast respond to Cys-SSH. Cys-SSH was added at 10 s, and then the fluorescent intensity decreased at 797 nm and increased at 749 nm during 10 - 70 s (Fig. S5a). The ratio-fluorescence signal response to Cys-SSH changes would reach saturation within 60 s (Fig. S5b), indicating that our probe could be used as a unique real-time bioimaging tool for intracellular Cys-SSH.

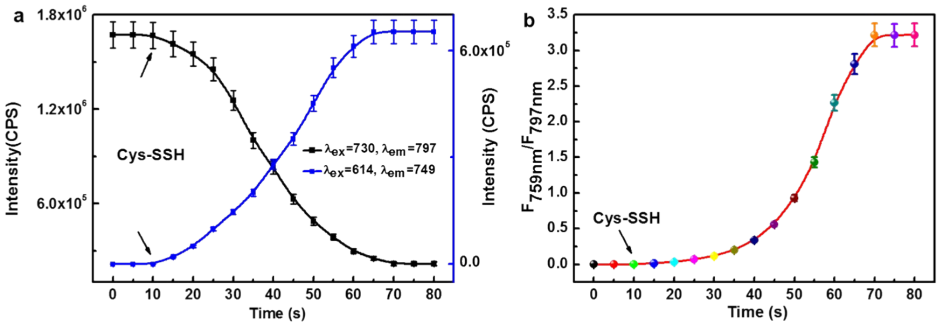

Fig. S5 Time dependent fluorescent ratio ($F_{749\ nm}/F_{797\ nm}$) of probe Cy-Dise (10 μM) towards Cys-SSH (20 μM) during 0 - 80 s. Cys-SSH was added at the reaction time of 10 s in 10 mM HEPES buffer (pH 7.4). The reactions were measured during 0 - 80 s at 37 ^oC. $F_{797\ nm}$: λ_{ex} = 730 nm, λ_{em} = 797 nm; $F_{749\ nm}$: λ_{ex} = 614 nm, λ_{em} = 749 nm.

8. Reaction Mechanism Discussion between Probe Cy-Dise and Cys-SSH

We had described the reaction mechanism in Fig. 1, the reduction of diselenide immediately formed an intermediate. Then a fast intramolecular cyclization occurred by cleavage of neighboring carbamate bond to release fluorophore. This proposed reaction mechanism was verified by HRMS, as shown in Fig. S6. The reaction intermediate HRMS (ESI$^+$): m/z $C_{59}H_{77}N_6O_{11}Se^+$ calcd. 1125.4815, found [M$^+$] 1125.4816. The reaction product HRMS (ESI$^+$): m/z $C_{56}H_{73}N_6O_9^+$ calcd. 973.5435, found [M$^+$] 973.5435.

However, there is a possibility which should be addressed, that is, the reduction of diselenide may formed an intermediate **Cy-a** (Fig. S7). However, we did not detect intermediate **Cy-a** by HRMR. Maybe intermediate **Cy-a** was high reactivity or unstable. Nonetheless, the intermediate selenylsulfides of **Cy-a** could be reduced by Cys-SSH straightway to form **Cy-se**.[28] Then a fast intramolecular cyclization occurs by cleavage of neighboring carbamate bond to release fluorophore. Even if our probe might follow the steps according to Fig. S7, there was also no extra consumption by the reaction.

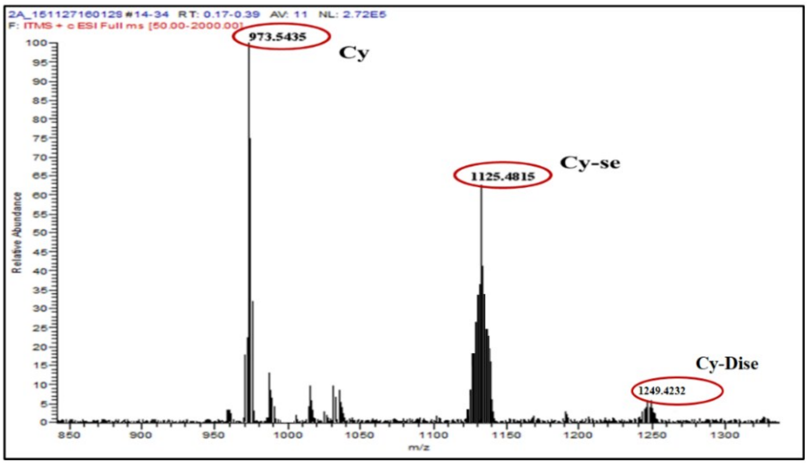

Fig. S6 HRMS of reactant Cy-Dise, the reaction intermediate Cy-se and the reaction product Cy.

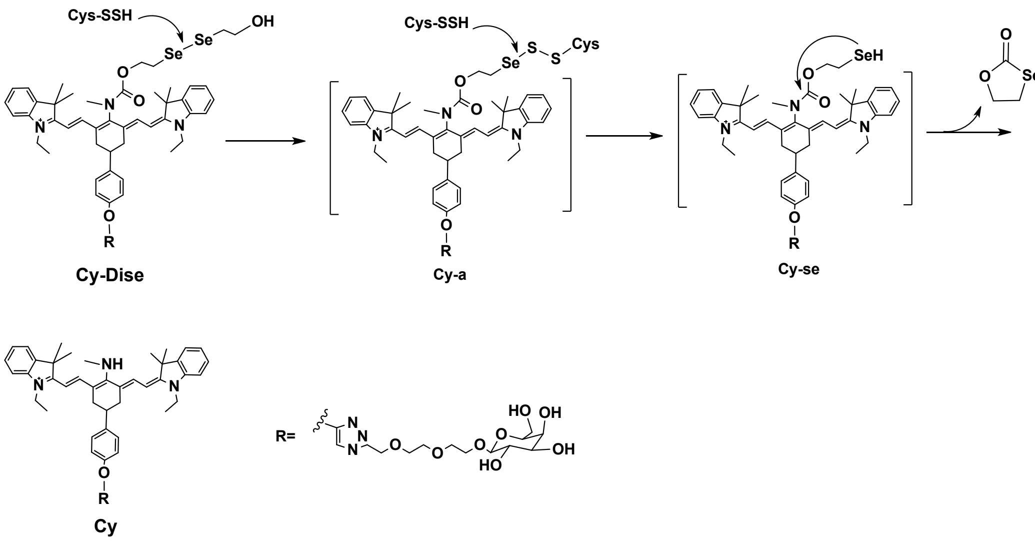

Fig. S7 The possible reaction mechanism between probe Cy-Dise and Cys-SSH.

9. MTT assay for Cy-Dise

To access the potential toxicity of **Cy-Dise**, MTT assays were carried out. HepG2 cells (10^6 cells/mL), HL-7702 cells (10^6 cells/mL) and primary mouse hepatocytes (10^6 cells/mL) were planted into 96-well microtiter plates in DMEM or RPMI 1640 with 10% fetal bovine serum (FBS). Plates were maintained at 37 °C in a 5% CO_2/95% air incubator for 24 h. Then the cells were incubated for 24 h at 37 °C in a 5% CO_2/95% air upon different concentrations probe of 0 μM to 100 μM respectively. MTT solution (5.0 mg/mL, PBS) was then added to each well. After 4 h, the remaining MTT solution was removed, and 200 μL of DMSO was added to each well, shaking 10 min to dissolve the formazan crystals at room temperature. Absorbance was measured at 570 nm and 630 nm in a TECAN infinite M200pro microplate reader.

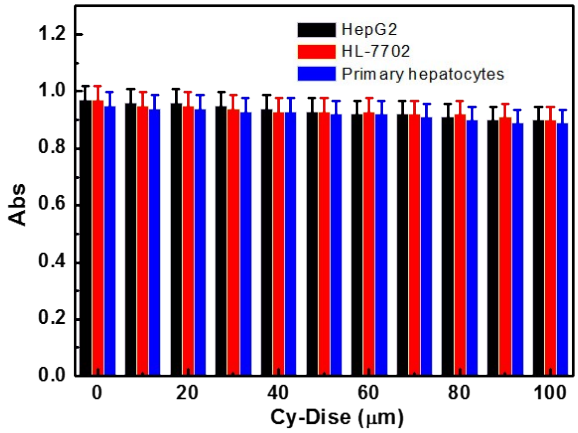

Fig. S8 MTT assay of HepG2, HL-7702 cells and primary hepatocytes with different concentrations of Cy-Dise. The experiment was repeated three times and the data were shown as mean (±S.D.).

10. Sublocation in Cells

To establish applicability of **Cy-Dise** in subcellular studies, we performed the co-localization experiments using **Cy-Dise** (10 μM), Calcein-AM, (1 μg/mL) (a commercial cytoplasm marker) and a DNA marker Hoechst 33342 (1 μg/mL) (a commercial nuclear dye) to determine the co-localization of fluorescence emission. The cells were incubated with Hoechst 33342 for 30 min, 1 μM Calcein-AM for 10 min and **Cy-Dise** for 5 min subsequently. With the help of Image-Pro Plus software, the respective spectra acquired from the three dyes were shown in Fig.

S9. The fluorescent images of **Cy-Dise** (red channel) and Calcein-AM (green channel) merged primely. Hoechst 33342 exhibited a perfect sublocation in nucles. The results confirmed that our probe could specifically localize in cytoplasm. And our probe also indicated a potentially powerful approach for real-time imaging cytoplasm Cys-SSH changes in cells and *in vivo*.

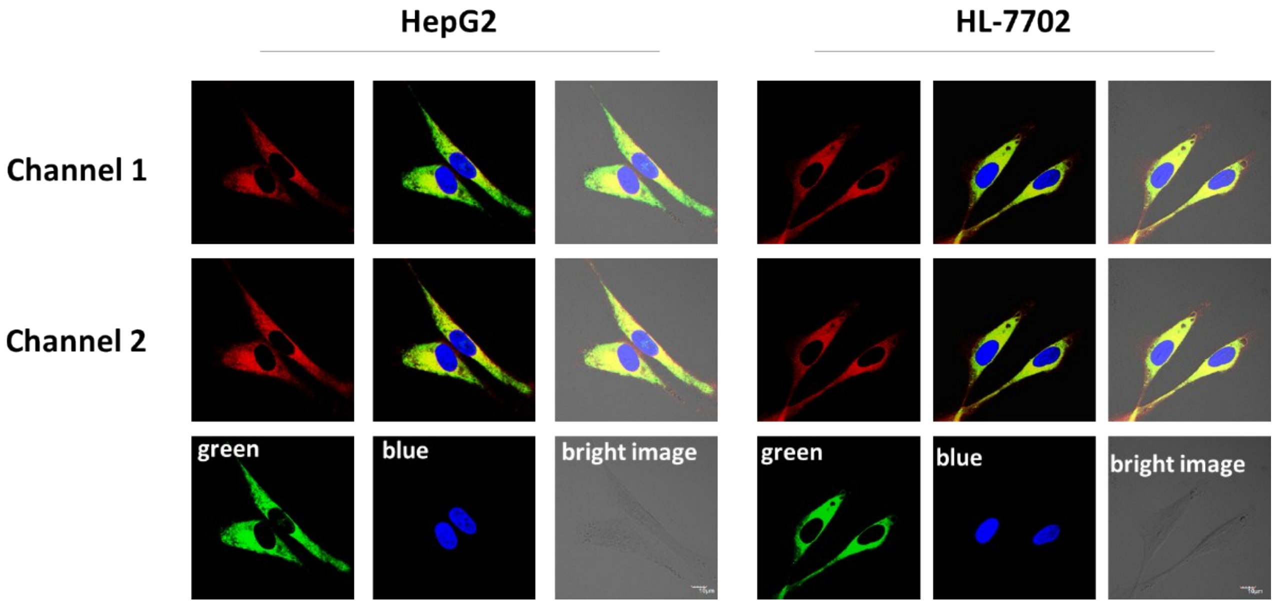

Fig. S9 Confocal microscopy images of live HepG2 and HL-7702 costained by Cy-Dise (10 μM), Calcein-AM, (1 μg/mL) and Hoechst 33342 (1 μg/mL). The cells were incubated with Hoechst 33342 for 30 min, 1 μM Calcein-AM for 10 min and then **Cy-Dise** for 5 min at 37 °C subsequently and imaged with **Cy-Dise** (channel 1: λ_{ex} = 730 nm, λ_{em} = 750 - 800 nm and channel 2: λ_{ex} = 635 nm, λ_{em} = 690 - 740 nm), Calcein-AM (green channel: λ_{ex} = 488 nm, λ_{em} = 500 - 550 nm) and Hoechst 33342 (blue channel: λ_{ex} = 405 nm, λ_{em} = 425 - 525 nm). Florescent images of HepG2, HL-7702 were acquired on Olympus FV1000 confocal laser-scanning microscope with an objective lens (× 60). Scale bar: 10 μm.

11. Plots of Relative Fluorescence Intensities of Cy-Dise against Time for Fig. 4A and Fig. 4B

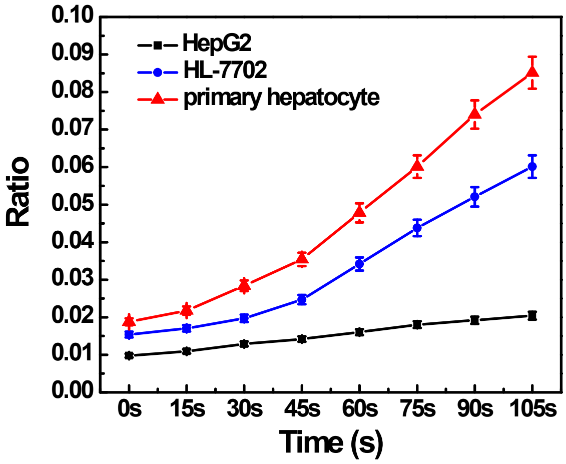

Fig. S10 Plots of average ratio intensities of Cy-Dise against time for Fig. 4A and Fig. 4B. The experiment was repeated three times and the data are shown as mean (±S.D.).

12. Flow Cytometry Analysis for Fig. 4A and Fig. 4B

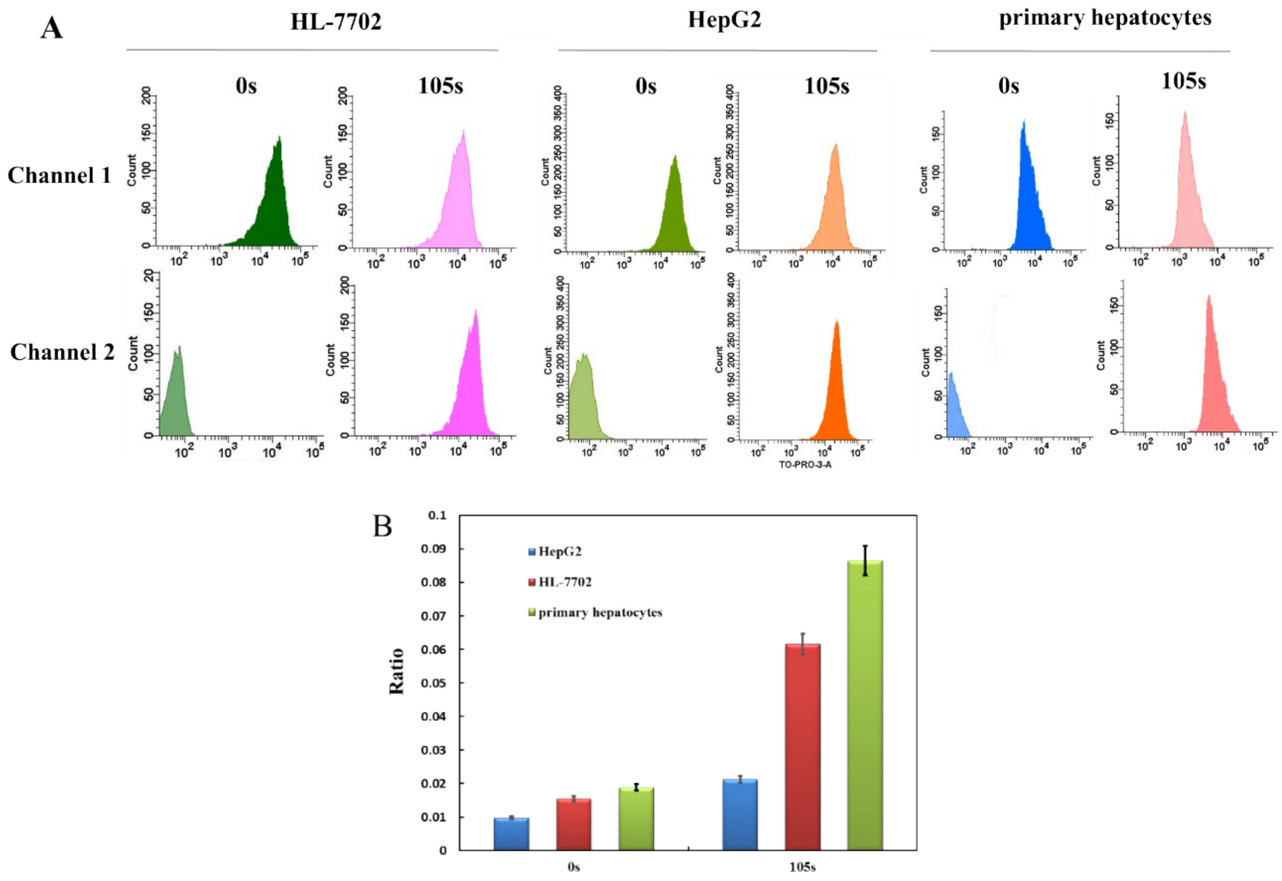

Fig. S11 Flow cytometry analysis A) and corresponding mean ratio intensity B) for Fig. 4A and Fig. 4B. The experiment were repeated three times and the data were shown as mean (±S.D.).

13. Flow Cytometry Analysis for Fig. 4C

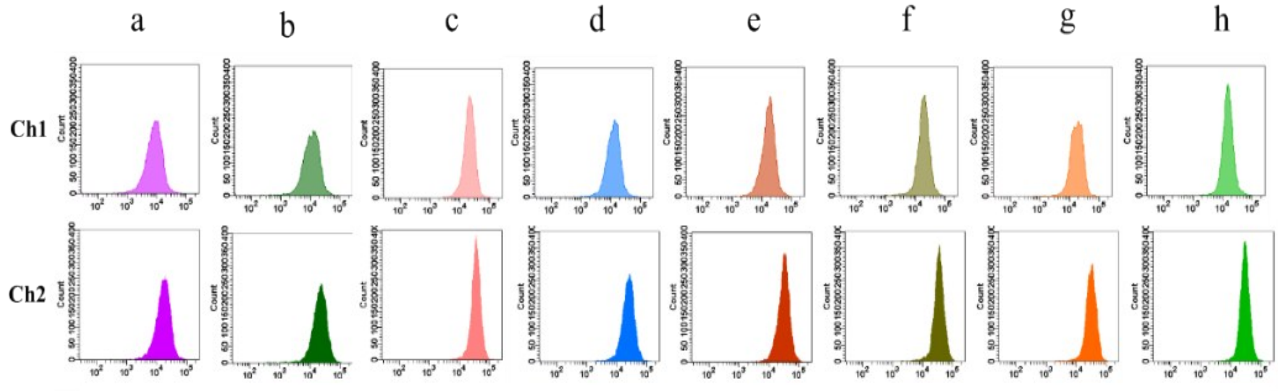

Fig. S12 Flow cytometry analysis for Fig. 4C.

14. Bright-field Images of Fig. 4B

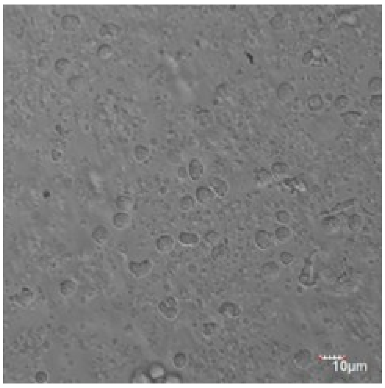

Fig. S13 Bright-field images of Fig. 4B. Scale bar: 10 μm.

15. Bright-field Images of Fig. 4C

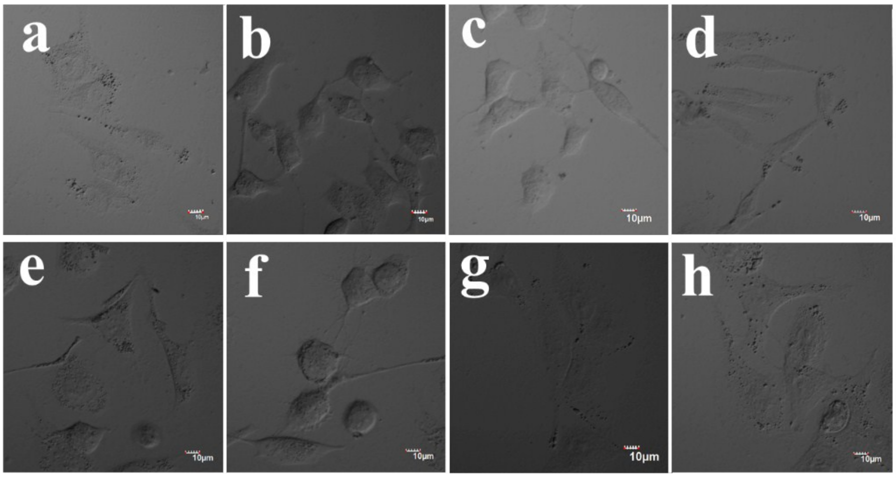

Fig. S14 Bright-field images of Fig. 4C. a) Bright-field image of Fig. 4C-a. b) Bright-field image of Fig. 4C-b. c) Bright-field image of Fig. 4C-c. d) Bright-field image of Fig. 4C-d. e) Bright-field image of Fig. 4C-e. f) Bright-field image of Fig. 4C-f. g) Bright-field image of Fig. 4C-g. h) Bright-field image of Fig. 4C-h. Scale bar: 10 μm.

16. Ratio Images of Time-dependent Cys-SSH Generation in NEM Treated Primary Hepatocytes

In order to verify that the probe could selectively image Cys-SSH generation in primary hepatocytes, NEM treated primary hepatocytes were used to performed as control. The primary hepatocytes were pretreated with *N*-ethylmaleimide (NEM) to deplete all the endogenous Cys-SSH. As shown in Fig. S15, there was nearly no ratiometric fluorescence signal observed. This indicated that probe **Cy-Dise** possessed good selectivity towards Cys-SSH.

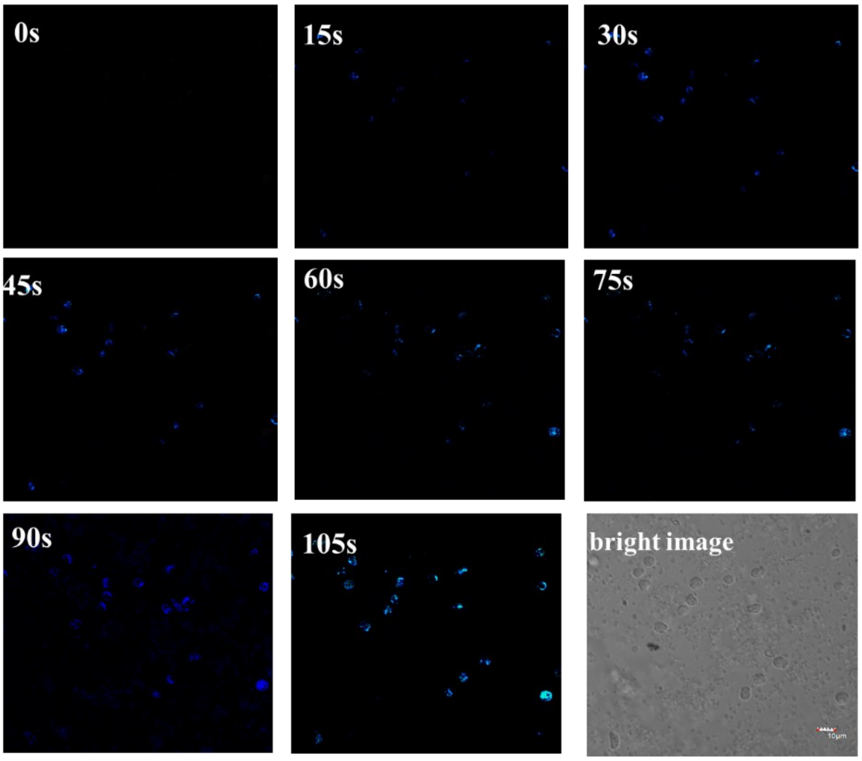

Fig. S15 Time dependent ratio images of endogenous Cys-SSH generation in NEM treated primary hepatocytes. Cells were cultured with 5 mM NEM for 30 min, then incubated with 10 μM Cy-Dise for 5 min, and then ratio images were recorded at different time points: 0 s, 15 s, 30 s, 45 s, 60 s, 75 s, 90 s, 105 s. Pseudo-color ratio images indicate the ratio of channel 2 *vs* channel 1 at the same time point. Fluorescent collection window for channel 1: 750-800 nm (λ_{ex} = 730 nm); channel 2: 690-740 nm (λ_{ex} = 635 nm). Scale bar: 10 μm.

17. LC/MS Analysis for Cys-SSH in Cell Lysates

In order to get the accurate concentration of Cys-SSH in HL-7702, HepG2 cells and primary hepatocytes, we exploited Tag-Switch assay based on the LC/MS analysis.[19] Firstly, standard Cys-SS-bimane was synthesized by the reaction between 0.5 mM Cys, 0.5 mM P-NONOate and 0.5 mM NaHS in 10 mM Tris-HCl (pH 7.4) at room temperature for 30 min. Then 5 mM Br-bimane was added to the reaction mixture at room temperature for 30 min. The synthesized Cys-SS-bimane was purified by RP-HPLC. CysSS-bimane, MS (ESI, positive): 344.0 $[M+H]^+$.

The cultured HepG2, HL-7702 cells and primary hepatocytes were washed with PBS buffer twice. After centrifugation and removal of supernatant liquid, the resulting cells were stored at -80°C for 5 min. After thawing to room temperature, the cells were lysed by hyperacoustic in 1 mL lysis buffer (PBS with 0.1% Triton X-100, pH 7.4) and the supernatant liquid was collected after centrifugation (10,000 × *g*, 10 min, 4 °C). Then 100 μL cell lysates were mixture with 500 μL of a methanol solution containing 5 mM Br-bimane. The mixture was incubated at 37 °C for 30 min. After centrifugation (10,000 × g, 10 min, 4 °C), supernatants were collected to perform LC-MS/MS analysis with synthesized Cys-SS-bimane as standard.

Analytical LC-MS/MS data for Cys-SSH in the cell lysis samples were acquired by LC-ESI-MS/MS. Samples were separated on RP-HPLC with a YMC-Triart C18 column (50 × 2.0 mm inner diameter), with a linear 5% - 90% methanol gradient for 14 min in 0.1% formic acid at 40 °C. Polysulfide derivatives were identified and quantified by means of MRM as shown in Fig. S16, with precursor ion (m/z): 344, product ion (m/z): 192, fragmentor voltage: 90 v, CID: 13. Quantification of Cys-SS-bimane in cell lysates was shown in Table 1.

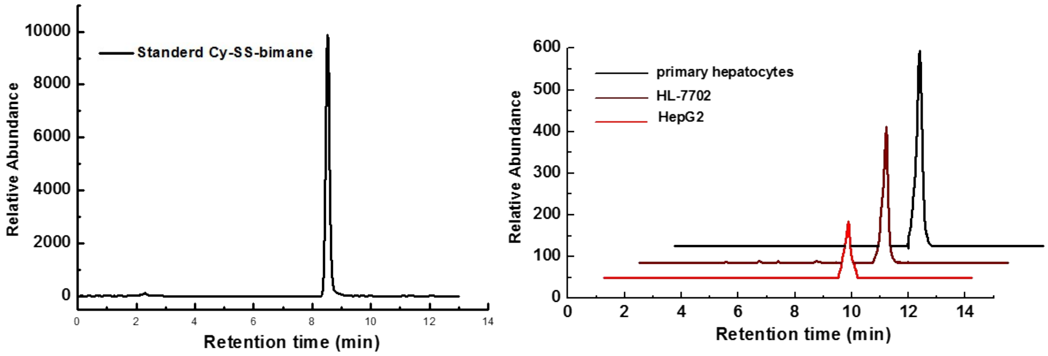

Fig. S16 LC-MS/MS analysis of Cys-SS-bimane in cell lysates.

Table 1. Quantification of Cys-SS-bimane in cell lysates.

Analyte	Concentration
**Standerd Cys-SS-bimane**	55 μM
**HepG2 cells lysates**	0.88 ± 0.2 μM
**HL-7702 cells lysates**	1.94 ± 0.4 μM
**Primary hepatocytes lysates**	2.58 ± 0.3 μM

18. H&E (hematoxylin and eosin) Staining of the Liver of Normal Rat and Hepatic Carcinoma Rat

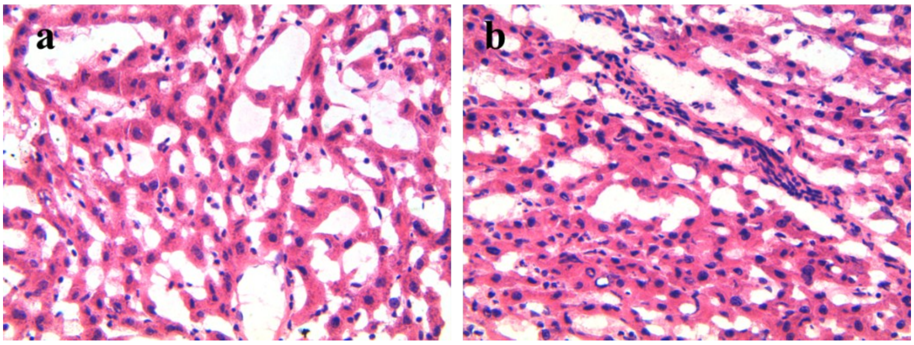

Fig. S17 H&E staining of the liver of normal rats a) and hepatic carcinoma rats b). × 400

19. *In vivo* and *ex vivo* imaging of Cys-SSH in Walker-256 tumor SD rats by control probe.

In order to further verify the liver targeting ability of the probe, a control probe (Cy$_3$-Dise) without liver targeting group was designed and synthesized. As shown in fig. S18, compound **3** (63.7 mg, 0.1 mmol) and triphosgene (0.09 g, 0.3 mmol) were dissolved in 50 mL anhydrous CH_2Cl_2 under Ar atmosphere.[1] The mixture was suspended at 0 °C, then 1 mL DIPEA was added. The reaction was lasted for 30 min, and the color of the solution changed into green from blue. After removed solvent in vacuum, the obtained residue was dissolved in 50 mL anhydrous CH_2Cl_2, and added DIPEA (1 mL) and DMAP (20 mg). Compound **8**, (0.050 g, 0.2 mmol) in CH_2Cl_2 (2 mL) was added into above mixture, then the reaction mixture was stirred at 25°C, TLC monitored the reaction until the starting material was completely consumed. The obtained solid residue was purified through a silica gel chromatography (200 - 300 mesh) with gradient eluent of CH_2Cl_2 and CH_3OH (100:0 to 85:15 v/v). The solvent was removed in vacuum, and the obtained solid was dissolved in CH_2Cl_2, filtered and evaporated to give a green solid (79 mg, yield 87 %). ^1H NMR (500 MHz, $CDCl_3$-D_1) δ (ppm): 7.74-7.68 (q, 2H), 7.43-7.35 (m, 6H),

7.29-7.20 (m, 5H), 7.08-7.00 (m, 3H), 6.15-6.06 (m, 2H), 5.32 (s, 2H), 4.75-472, (m, 2H), 4.45-4.43 (m, 8H), 2.91 (s, 1H), 1.76-1.65, (m, 12H), 1.45-1.39 (m, 6H), 1.28-1.21 (m, 9H). ^{13}C NMR (125 MHz, $CDCl_3$-D_1) δ (ppm): 171.24, 156.33, 156.29, 156.22, 141.26, 140.69, 129.56, 128.74, 128.70, 128.06, 125.28, 125.25, 122.05, 114.93, 110.55, 103.62, 103.57, 71.81, 71.73, 70.41, 64.27, 61.56, 61.39, 60.98, 55.61, 53.30, 49.01, 47.25, 37.41, 36.48, 33.95, 32.81, 32.32, 29.52, 29.11, 28.95, 28.17, 27.74, 22.07, 20.76, 20.31, 15.64, 14.80, 12.24. LC-MS (ESI^+): m/z $C_{49}H_{58}N_3O_4Se_2^+$ calcd. 912.2752, found [M^+] 912.2753.

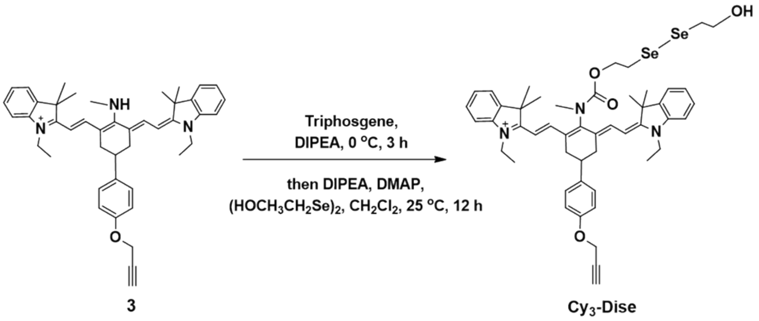

Fig. S18 Synthetic routes for control probe Cy_3-Dise.

Then the control probe was carried out to perform the *in vivo* and *ex vivo* imaging of Cys-SSH in Walker-256 tumor SD rats. The fluorescence images were obtained from two fluorescence collection windows. Channel 1: λ_{ex} = 730 nm filter: 780 nm, and channel 2: λ_{ex} = 610 nm filter: 710 nm. The Walker-256 tumor SD rats were given intravenous injection of the control probe (10 μM, 50 μL, in 1:99 DMSO/saline, v/v) for 15 min. As shown in Fig. S19, the in vivo imaging of Walker-256 tumor SD rats in channel 1 by control probe showed almost whole body range fluorescence signal, because the control probe was widely distributed through the blood circulation. And from the *ex vivo* imaging, some other organs besides liver, such as spleen and kidney also gathered the control probe. The results illustrated that the control probe was poor liver targeting effect and the liver targeting group was indispensable. And in channel 2, the *in vivo* imaging showed fluorescence signal around liver position and the *ex vivo* imaging performed the fluorescence enhancement in liver, which may be due to the higher Cys-SSH concentration in liver than other organs. From the *in vivo* and *ex vivo* imaging of Cys-SSH in Walker-256 tumor SD rats by the control probe, we conclude that the liver targeting group was indispensable and absolutely necessary.

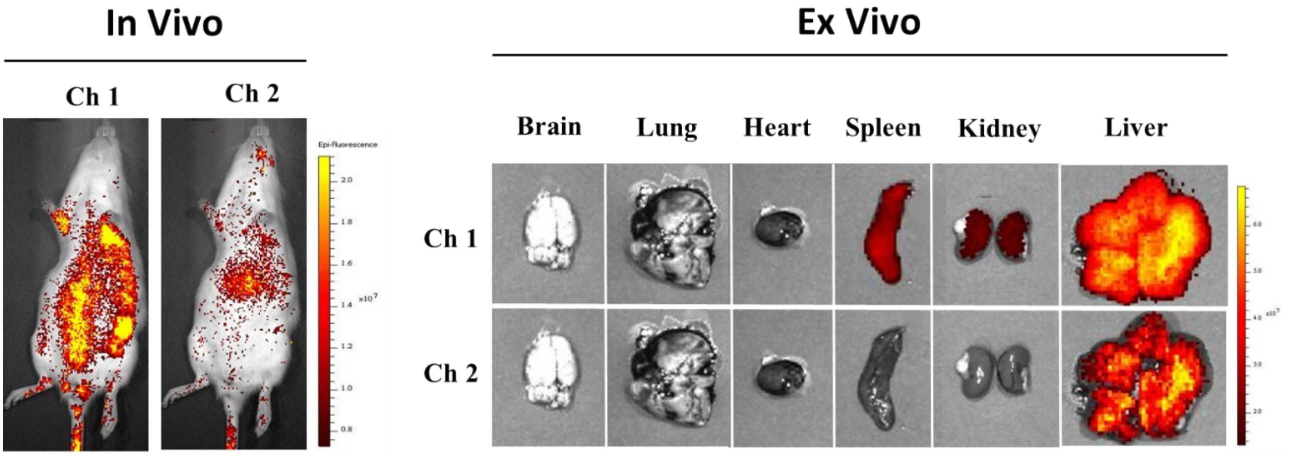

Fig. S19 *In vivo* and *ex vivo* imaging of Cys-SSH by intravenous injection of control probe in Walker-256 tumor SD rats. *In vivo*: Walker-256 tumor SD rats were injected with control probe (10 μM, 50 μL, in 1:99 DMSO/saline, v/v) for 15 min. *Ex vivo*: imaging of Cys-SSH in organs sacrificed from the Walker-256 tumor SD rats. The experiments were repeated three times and the data were shown as mean (±S.D.).

20. Supplementary Movie 1

Miniature video of time dependent ratio images ($F_{channel\ 2}/F_{channel\ 1}$) of endogenous Cys-SSH in HL-7702 cells during 0 - 105 s. Pseudo-color ratio images indicate the ratio of channel 2 *vs* channel 1 at the same time point. Fluorescent collection window for channel 1: 750 - 800 nm (λ_{ex} = 730 nm); channel 2: 690 - 740 nm (λ_{ex} = 635 nm). Scale bar =10 μm.

21. Supplementary Movie 2

Miniature video of time dependent ratio images ($F_{channel\ 2}/F_{channel\ 1}$) of endogenous Cys-SSH in HepG2 cells during 0 - 105 s. Pseudo-color ratio images indicate the ratio of channel 2 *vs* channel 1 at the same time point. Fluorescent collection window for channel 1: 750 - 800 nm (λ_{ex} = 730 nm); channel 2: 690 - 740 nm (λ_{ex} = 635 nm). Scale bar =10 μm.

22. ^{1}H NMR, ^{13}C NMR and HRMS of Probe Cy-Dise

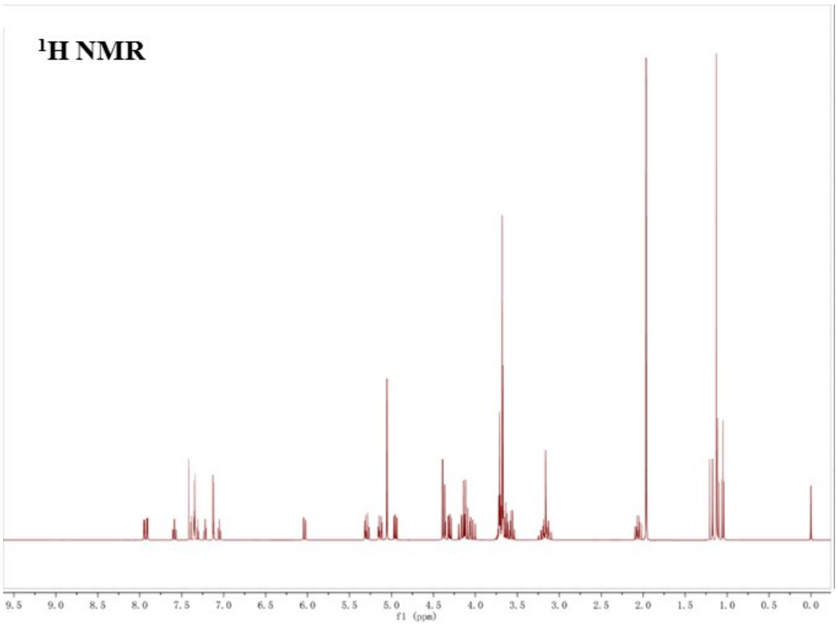

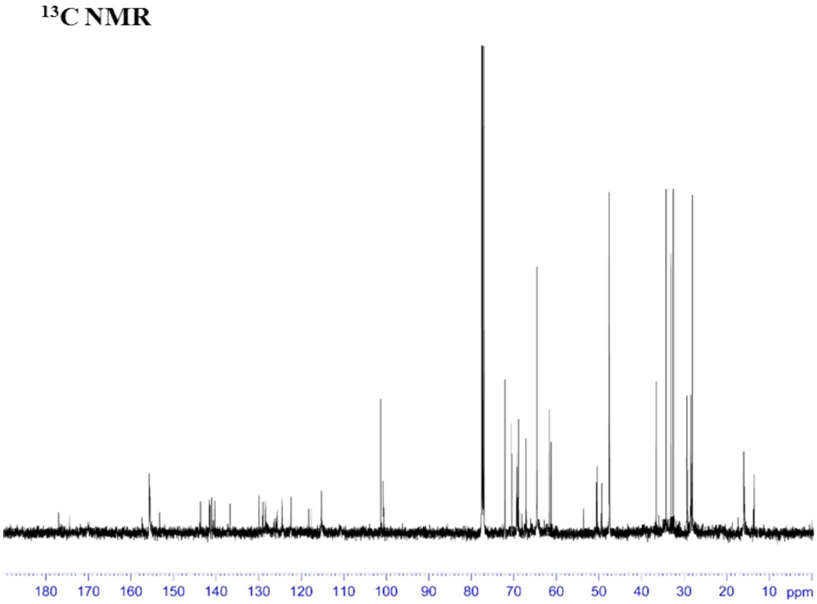

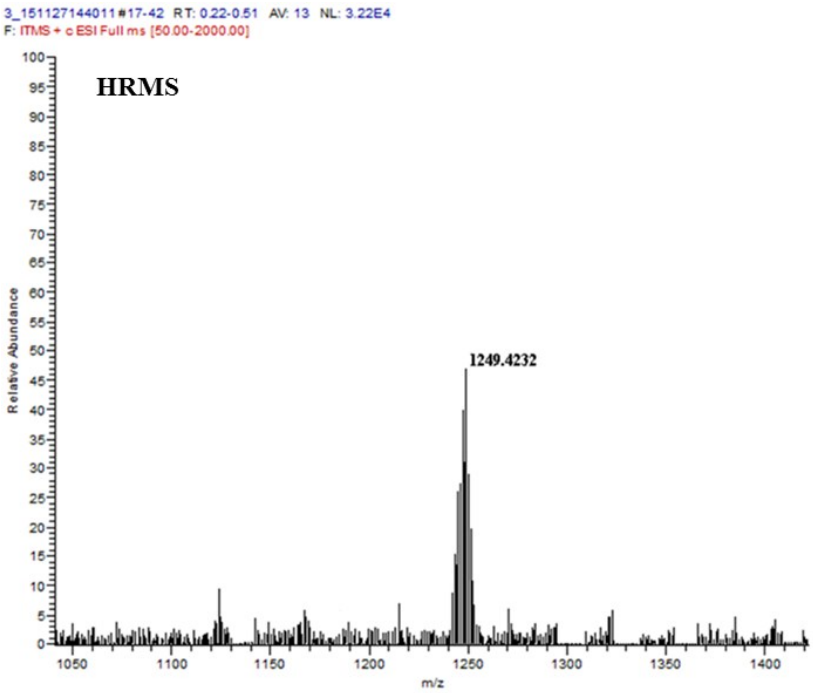

23. References

(1) D. Cavallini, C. De Marcoc and B. Mondovi, *Arch. Biochem. Biophys*., 1960, **87**, 281-288.

(2) G. Jansen, J. van der Heijden, R. Oerlemans, W. F. Lems, I. Ifergan, R. J. Scheper, Y. G. Assaraf and B. A. Dijkmans, *Arthritis Rheum.*, 2004, **50**, 2130-2139.

(3) J. A. Terao, A. Nagao, D. K. Park and B. P. Lim, *Meth. Enzymol.*, 1992, **213**, 454-460.

(4) J. C. Morris, *J. Phys. Chem.*, 1966, **70**, 3798-3805.

(5) V. Massey, and D. J. Edmondson, *Biol. Chem.*, 1970, **245**, 6595-6598.

(6) A. L. Nieminen, A. M. Byrne, B. Herman and J. J. Lemasters, *Am. J. Physiol.*, 1997, **272**, 1286-1294.

(7) R. M. Uppu, *Anal. Biochem.*, 2006, **354**, 165-168.

(8) H. Maeda, K. Yamamoto, Y. Nomura, I. Kohno, L. Hafsi, N. Ueda, S. Yoshida, M. Fukuda, Y. Fukuyasu, Y. Yamauchi and N. Itoh, *J. Am. Chem. Soc.*, 2005, **127**, 68-69.

(9) T. W. Hart, *Tetrahedron. Lett.*, 1985, **26**, 2013-2016.

(10) I. Artaud and E. Galardon, *Chembiochem.*, 2014, **15**, 2361-2364.

(11) T. H. Poole, J. A. Reisz, W. Zhao, L. B. Poole, C. M. Furdui and S. B. King, *J. Am. Chem. Soc.*, 2014, **136**, 6167-6170.

(12) J. Pan and K. S. Carroll, *ACS Chem. Biol.*, 2013, **8**, 1110-1116.

(13) C. E. Paulsen and K.S Carroll, *Chem. Biol.* 2009, **16**, 217-225.

(14) (a) E. Cuevasanta, M. Lange, J. Bonanata, E. L. Coitiño, G. Ferrer-Sueta, M. R. Filipovic and B. Alvarez, *J. Biol. Chem.* 2015, **290**, 26866-26880; (b) É. Dóka, I. Pader, A. Bíró, K. Johansson, Q. Cheng, K. Ballagó, J. R. Prigge, D. Pastor-Flores, T. P. Dick, E. E. Schmidt, E. S. Arnér and P. Nagy, *Sci. Adv.* 2016, **2**, DOI: 10.1126/sciadv.1500968.

(15) T. Takata, D. Saeki, Y. Makita, N. Yamada and N. Kihara, *Inorg. Chem.* 2003, **42**, 3712-3714.

(16) W. Chen, C. Liu, B. Peng, Y. Zhao, A. Pacheco and M. Xian, *Chem. Sci.* 2013, **4**, 2892-2896.

(17) P. O. Seglen, *Methods Cell Biol.*, 1976, **13**, 29-83.

(18) T. Hoshiba, C. S. Cho, A. Murakawa, Y. Okahata and T. Akaike, *Biomaterials*, 2006, **27**, 4519-4528.

(19) T. Ida, T. Sawa, H. Ihara, Y. Tsuchiya, Y. Watanabe, Y. Kumagai, M. Suematsu, H. Motohashi, S. Fujii, T.

Matsunaga, M. Yamamoto, K. Ono, N. O. Devarie-Baez, M. Xian, J. M. Fukuto and T. Akaike, *Proc. Natl. Acad. Sci. U. S. A.*, 2014, **111**, 7606-7611.

(20) R. Yang, F. J. Rescorla, C. R. Reilly, P. R. Faught, N. T. Sanghvi, L. Lumeng, T. D. Jr Franklin and J. L. Grosfeld, *J. Surg. Res*., 1992, **52**, 193-198.

(21) T. Sun, Y. Jin, R. Qi, S. Peng and B. Fan, *Macromol. Chem. Phys.*, 2013, **214**, 2875−2881.

(22) S. Y. Oh, R. Ezaki, K. Akagi and H. Shirakawa, *J. Polym. Sci., Part A: Polym. Chem.*, 1993, **31**, 2977.

(23) Z. Yang, J. H. Lee, H. M. Jeon, J. H. Han, N. Park, Y. He, H. Lee, K. S. Hong, C. Kang and J. S. Kim, *J. Am. Chem. Soc.*, 2013, **135**, 11657-11662.

(24) N. Narayanan and G. Patonay, *J. Org. Chem.*, 1995, **60**, 2391-2395.

(25) R. Wang, L. Chen, P. Liu, Q. Zhang and Y. Wang, *Chem.-Eur. J.*, 2012, **18**, 11343-11349.

(26) F. Amblard, J. H.Cho and R. F. Schinazi, *Chem. Rev.*, 2009, **109**, 4207-4220.

(27) M. H. Lee, H. M. Jeon, J. H. Han, N. Park, C. Kang, J. L. Sessler and J. S. Kim, *J. Am. Chem. Soc.*, 2014, **136**, 8430-8437.

(28) D. Steinmann, T. Nauser and W. H. Koppenol, *J. Org. Chem.*, 2010, **75**, 6696-6699.
